# The evolution of nanopore measurements: from biological out-of-plane pores to plastic in-plane pores

**DOI:** 10.1039/d5lc00885a

**Published:** 2026-01-28

**Authors:** Khurshed Akabirov, Hanna Nguyen, Shakila Peli Thanthri, Sheila M. Barros, Maximillian Chibuike, Sunggook Park, Steven A. Soper

**Affiliations:** a Department of Chemistry, The University of Kansas Lawrence KS 66045 USA; b Center of BioModular Multiscale Systems for Precision Medicine USA; c Department of Industrial and Mechanical Engineering, Louisiana State University Baton Rouge LA 70803 USA; d Department of Mechanical Engineering, The University of Kansas Lawrence KS 66045 USA; e Bioengineering Program, The University of Kansas Lawrence KS 66045 USA; f KU Cancer Center, University of Kansas Medical Center Kansas City KS 66160 USA ssoper@ku.edu; g Kansas Institute of Precision Medicine Kansas City KS 66160 USA

## Abstract

Nanopore sensing provides an ideal strategy for the label-free detection of single molecules in a variety of application scenarios. Working under the principle of resistive pulse sensing (RPS), nanopores consist of constrictions with sub-100 nm dimensions to enable single-molecule resolution by matching pore size to target dimensions (scaling); the optimal signal-to-noise ratio (SNR) results when the electrically biased pore is comparable in size to the molecule to be analyzed. When single molecules are electrokinetically transported through such remarkably small pores, they temporarily disturb the flux of ions moving through them, generating unique signals. These signals vary based upon the molecules' shape, size, orientation, and other physicochemical properties. Nanopores are generally divided into two main categories owing to their fabrication approach and material: biological and solid state. While biological nanopores have been the dominant sensor format due to their exceptionally small size, solid-state nanopores can demonstrate high performance characteristics attributed to their rigidity, stability, and high versatility in shape, material, and configuration. This review will explore the state-of-the-art in biological and solid-state nanopores and their abilities to detect and identify single biomolecules in a label-free manner. We will also review two topographical configurations of nanopore sensors; in-plane and out-of-plane sensors. The evolution of nanopore sensing will be reviewed, starting with out-of-plane biological sensors and progressing to in-plane sensors fabricated in plastics *via* replication technologies.

## Abstract 2

A comprehensive review of in-plane and out-of-plane nanopore configurations for label-free single molecule detection is discussed herein. Also reviewed is a description of varied materials for both nanopore configurations and effects on target SNR.

## Graphical abstract

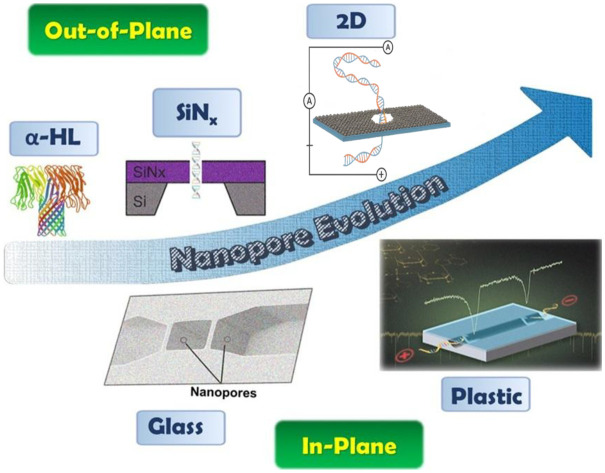

## Introduction

Resistive pulse sensing (RPS) uses an electrically biased nano-constriction (*i.e.*, nanopore) with the monitoring of ionic current as individual entities translocate through the nanoscale pore. This technology traces its roots back to the pioneering work of Wallace H. Coulter, who developed the first “hole-based” sensing technique in the late 1940s and was later patented in 1953 as a method for detecting and counting micrometer-sized blood cells suspended in liquid (*i.e.*, Coulter counter).^1^ According to the Coulter principle,^2^ a particle moving through a constriction of similar size that separates two electrolyte-filled chambers produces a change in constriction resistance that is proportional to the volume of the particle translocating through the pore. While its initial development arose from the need for counting blood cells, DeBlois *et al.*^3^ adapted the technology about two decades later for the detection of colloidal particles and bacteriophages using sub-micrometer sized pores. Reducing the constriction size improved resolution and enabled the detection of individual biomolecules, thereby paving the way for new applications with major advancements occurring in the 1990s through efforts in single-molecule DNA sequencing.^4–7^ Since then, nanopore sensing applications have expanded due to their single molecule sensitivity, label-free capability, remarkable versatility, and enhanced insights into nanoscale phenomena.^8^ These insights resulted from an amalgamation of multiple disciplines including chemistry, biology, physics, engineering, and materials science. Nanopore sensing applications now encompass a vast array of molecule types and particles such as mono-^9^ and oligonucleotides,^10^ proteins,^11–15^ carbohydrates,^16^ viruses,^17–21^ extracellular vesicles,^22–24^ and others.

Single entity occupation within a nanopore modulates the flow of ions either by physically obstructing the pore, which leads to a decrease in ionic current (current blockade), or by introducing a local enrichment of charge carriers such as counterions associated with highly charged entities, which can increase the ionic current. Because detection is dependent on ionic current blockade or enhancement, the translocation dynamics (*i.e.*, current change magnitude, duration, and signal shape) may be correlated to unique characteristics and behavior of the entity moving through the pore under the influence of an electric field. These include properties such as size,^19,25–27^ conformation,^11^ mechanical stability/deformation,^21,28^ surface charge,^29^ and/or electrophoretic mobility.^14,18,23,25^ The diameter of classical nanopore sensors range from 1–100 nm while those of extended nanopore sensors range from 100–1000 nm.^30^ The sensor's physical (size, shape, orientation, conductivity) and chemical (composition and surface functional groups) properties as well as run buffer conditions may be tailored to fit the application need.

In this manuscript, we will provide a timely review of the evolution of RPS nanopore sensors using different materials and configurations for the sensing of single molecules. We will also discuss the basic operating principles of nanopore sensing, including ionic current modulation, voltage-driven analyte translocation, and signal interpretation based on molecular properties. This will give the reader a fundamental basis from which performance comparisons can be made for pores of different configurations and/or materials.

## General operating considerations for RPS

The high surface area-to-volume ratio associated with nanopores elicit a significant contribution of surface properties on the transport dynamics of single molecules through the pores and their RPS signals,^8,32^ which are typically negligible at the microscale. These phenomena include, but are not limited to, electrical double layer (EDL) overlap, ion permselectivity, and ion concentration polarization. Because electrostatic forces are a major contributor to these phenomena, factors such as electrolyte type and concentration, pH, and voltage must be considered as well. Finally, pore size and shape can affect the resultant signal observed in RPS. These effects will be discussed briefly in the sections that follow.

### Surface charge effects

**Fig. 1:**
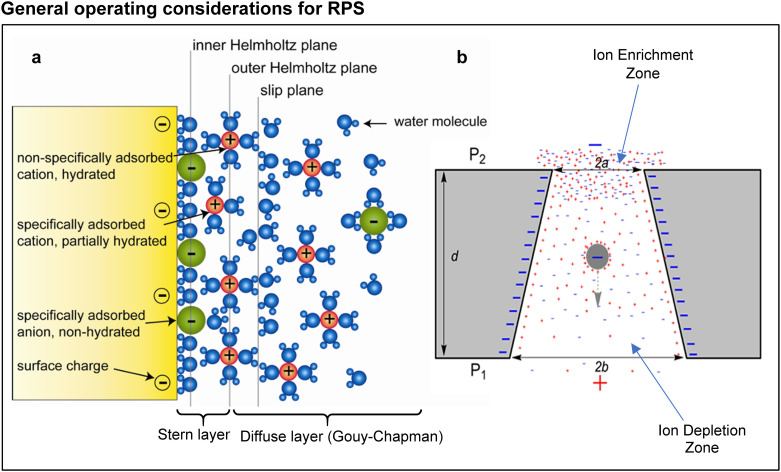
(a) The formation of electric double layer (EDL) on solid/liquid interface also known as Gouy–Chapman–Stern model. Negatively charged surface attracts/adsorbs non-hydrated anions, partially hydrated cations and fully hydrated cations forming inner Helmholtz (Stern layer) and outer Helmholtz (diffuse layer or Gouy–Chapman) planes. Diffuse layer mainly consists of hydrated co-ions. Reproduced with permission from Schoch *et al.*^31^ Copyright 2005 American Institute of Physics. (b) Ion enrichment (cathodic side) and depletion zones (anodic side) formed at a micro- to nanostructure interface.

Many surfaces acquire an electric charge when in contact with an electrolyte solution. The nature and density of this charge depend on a combination of material properties (surface functional groups), solution characteristics (pH and ionic species), and certain interactions (*e.g.*, adsorption/desorption at solid/liquid interfaces). This surface charge affects the distribution of hydrated counterions (ions of opposite charge to the surface charge) and co-ions in solution forming two distinct layers of ions known collectively as the EDL. Due to electrostatic interactions, a single layer of hydrated counterions is positioned adjacent to the surface forming the Stern layer (Fig. 1a). This fixed layer of counterions does not sufficiently screen the surface charge, so additional hydrated counterions accumulate next to the Stern layer.^31^ Because of their greater distance from the surface, these counterions are mobile due to Brownian motion. To maintain electrical neutrality, hydrated co-ions are also present. Together, these mobile counterions and co-ions form the diffuse layer where the concentration of counterions exceeds that of co-ions until it gradually approaches equilibrium with the bulk solution.

The interface between the Stern and diffuse layers is known as the slip or shear plane. The potential difference at this interface is called the zeta (*ζ*) potential. The *ζ* potential decays exponentially in the diffuse layer and the characteristic length over which this decay occurs is known as the Debye length (*λ*_D_). The Debye length is represented by;^8^1
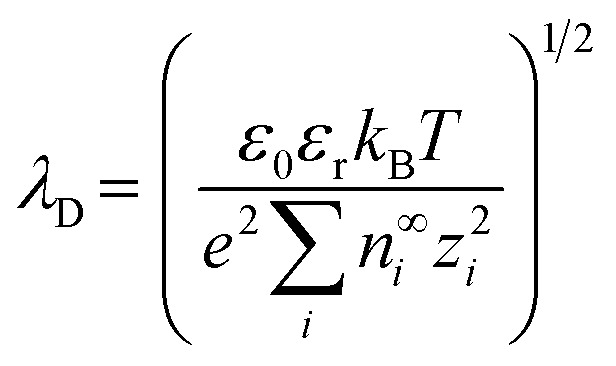
where *ε*_0_ is the vacuum permittivity, *ε*_r_ is the relative permittivity of the solution, *k*_B_ is the Boltzmann constant, *T* is absolute temperature, *e* is electron charge, *n*^∞^_*i*_ is the bulk volume density for the *i*th ion, and *z*_*i*_ is the charge of the *i*th ion. For a 1 : 1 electrolyte solution at 25 °C, eqn (1) simplifies to;^8^2
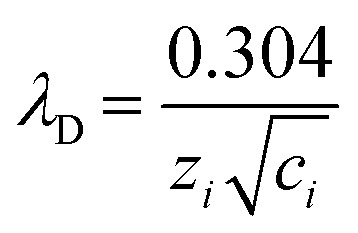
where *c*_*i*_ is the salt concentration. While surface charge density primarily affects the concentration and distribution of ions within the EDL, ionic strength directly influences its thickness. According to eqn (2), at bulk salt concentrations >100 mM, as is typical in RPS experiments, *λ*_D_ is <1 nm. For a historical basis of the EDL and mathematical derivations of *λ*_D_, readers are directed to a more in-depth review.^8^

Under a bias voltage, charged entities migrate electrophoretically towards electrodes of opposite charge. Neutral particles move with the bulk fluid flow, which is towards the cathode for negatively charged surfaces. This bulk flow, known as the electroosmotic flow (EOF), is generated from the viscous drag exerted by cations in the diffuse layer as they migrate towards the cathode. EOF can either reduce or increase the driving force on the molecule/particle depending on the direction of the electrophoretic mobility (*μ*_ep_). While particle charge density influences its *μ*_ep_, surface charge density and solution characteristics influence the electroosmotic mobility (*μ*_eof_), which can be described by eqn (3);^8^3
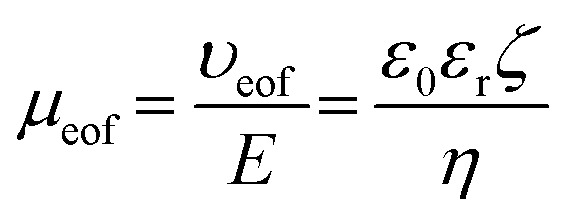
where *υ*_eof_ is the velocity of the EOF, *E* is the electric field strength, and *η* is the viscosity of the solution. The higher the surface charge density, the higher *μ*_eof_. Particle/molecule net movement, or apparent mobility (*μ*_app_), is determined by the vector sum of both *μ*_eof_ and *μ*_ep_.

When the dimension of the nanopore is comparable to that of the EDL, EDL overlap can occur transforming the classical plug-like EOF profile into a more parabolic flow profile.^33^ EDL overlap also causes the nanopore to become charge-selective, leading to ion current rectification (ICR) and concentration polarization.^32,34–36^ With EDL overlap, the ion flux must pass through the diffuse layer, which has a non-uniform distribution of ions despite being electrically neutral. This non-uniformity creates a potential gradient that favors the transport of counterions while hindering the movement of co-ions. When there is asymmetric surface charge and/or asymmetric nanopore geometry, ICR can occur stemming from the unequal migration of cations and anions that generate a higher ion current in one direction.^34,35,37,38^ This is observed as a deviation from ohmic behavior in the current–voltage (*I*–*V*) curve. The degree of rectification is determined from the rectification ratio (the ratio of current at voltages of opposite polarity but equal magnitude).^37^ Lower ionic strength solutions typically produce greater rectification because surface charge contributes more significantly to the total current. Because analyte-induced changes on the nanopore surface can modulate the *I*–*V* response, ICR can affect the accuracy of applications that rely on an Ohmic response.

Concentration polarization, while related to ICR, differs in its manifestation. It describes the spatial distribution of ions within and around a nanopore and can be induced by EDL overlap or by an electric field without requiring surface charge asymmetry.^39^ Ion transport involves both diffusion and electrokinetics. For negatively charged surfaces, when diffusive transport is slower than electrokinetic transport (*i.e.*, sufficiently high applied voltage), freely migrating cations concentrate on the cathodic side (enrichment zone) at a micro- to nanostructure interface leaving the anodic side depleted of cations (depletion zone); see Fig. 1b.^33,34,36^ Co-ions follow a similar enrichment and depletion pattern to preserve charge balance.

### Effects of voltage, salt, surface charge, and pore dimensions on RPS

Despite its well-established name, RPS is only a partial descriptor for the operational principles of the method because it monitors transient current changes rather than direct resistance. Current changes can result from blockades (resistive pulses), enhancements (conductive pulses),^40–42^ or a combination of both (biphasic pulses)^43,44^ depending on analyte and pore surface charge, buffer composition, and applied voltage. The baseline or open pore current (*I*_0_) is described by eqn (4),^45^ where *V* is the bias voltage, *μ*_+_ and *μ*_−_ represent the electrophoretic mobilities of cations and anions, respectively, *n*_±_ is the number density of the salt ions, *e* is the elementary charge, *h*_eff_ is the effective pore length or membrane thickness of the pore, and *d* is the diameter of the pore.4
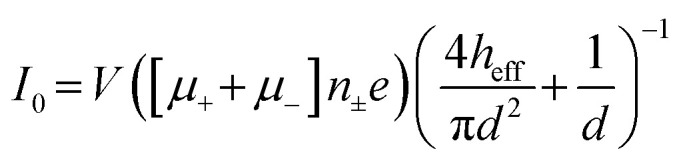
This expression shows that *I*_0_ scales with salt concentration, ion mobility, and pore geometry, making these parameters critical to signal optimization. In situations where surface charge is significant, EOF may also contribute to ion transport. The current change or pulse amplitude (Δ*I*) for a cylindrical pore at high ionic strengths (>100 mM) is described by eqn (5);^45^5
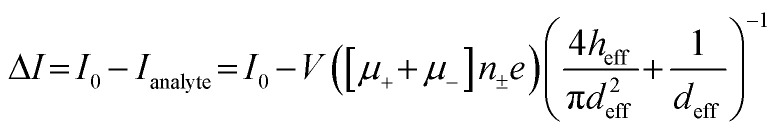
where *I*_analyte_ is the current during analyte occlusion of the pore and *d*_eff_ is the effective diameter of the analyte-occluded pore. Key factors affecting Δ*I* include voltage, salt type and concentration, pore and analyte surface charge, and their respective dimensions. Δ*I* scales with the hydrodynamic cross-section of the analyte but is inversely proportional to pore volume (effective sensing volume). Higher voltages increase *I*_0_ and Δ*I*, translocation speed through the pore (resulting in smaller pulse widths or dwell times, *t*_d_), and capture rate (the number of translocations per unit time). However, higher voltages also introduce more noise, particularly thermal (Johnson–Nyquist) noise due to increased agitation of ions and solvent molecules, which can reduce the SNR and affect measurement resolution.

**Fig. 2:**
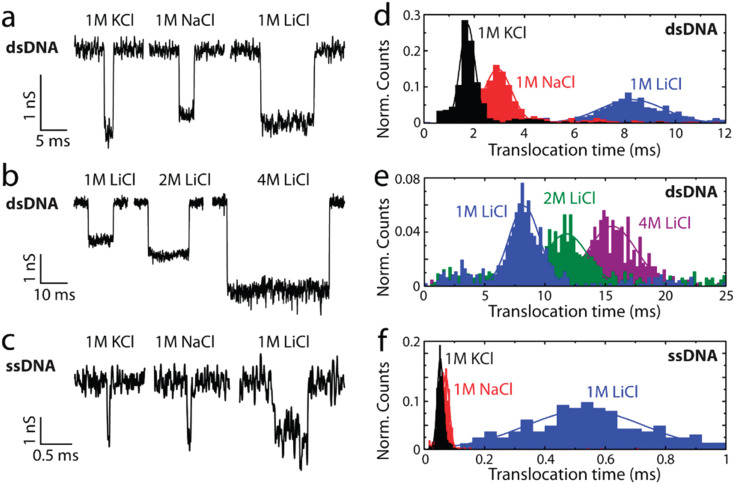
Effects of salt type on the RPS signal characteristics for both ssDNA and dsDNA analytes. (a) Representative current traces for 48.5 kbp λ-dsDNA translocations recorded at 5 kHz with different monovalent salts. (b) Similar recordings in 1 M, 2 M, and 4 M LiCl solutions. (c) Translocation traces for heat-denatured M13mp18 ssDNA in the presence of 8 M urea and 1 M monovalent salts, recorded at 30 kHz. (d–f) Corresponding histograms of translocation times for the conditions in (a–c). Reproduced with permission from Kowalczyk *et al.*,^46^ Copyright 2012 American Chemical Society.

Salt type influences Δ*I* through its conductivity (*e.g.*, K^+^ > Na^+^ > Li^+^); Fig. 2. While salt type weakly affects Δ*I*, it strongly impacts pulse duration *via* charge screening by counterions.^46^ Smaller, more strongly hydrated ions (Li^+^ < Na^+^ < K^+^) screen the surface charge more effectively, thereby reducing effective charge and increasing *t*_d_. Higher salt concentrations similarly affect pulse characteristics as they enhance charge screening. If charge reduction is such that *μ*_ep_ < *μ*_eof_, the translocation would be EOF-dominant.^15^ For proteins or protein-containing analytes, different salt types exert chaotropic (salting-out) or kosmotropic (salting-in) effects in accordance with the Hofmeister series.^15^ Asymmetric salt conditions, where the salt concentration is lower in the *cis* (analyte-loading) chamber relative to that in the *trans* (analyte-receiving) chamber, have been shown to increase event rates and pulse durations for negatively charged analytes.^47,48^ Wanunu *et al.*^47^ noted that the increased pumping of cations into the *cis* chamber creates a polarized environment near the pore. This polarization increases the potential well adjacent to the pore funneling analytes towards the entrance and enhancing their capture rate. For negatively charged analytes and surfaces, a higher *trans*-side salt concentration increases pulse durations due to an increased counterion flux to the *cis* side, which enhances EOF and reduces *μ*_app_. Additionally, a lower salt concentration results in a higher electrical resistance leading to a greater voltage drop outside the pore (*cis* side), and consequently a lower voltage drop inside the pore compared to symmetric salt conditions. This reduced effective voltage results in a weaker electric field slowing down analyte translocation through the nanopore.^47^

The non-specific nature of charge screening also affects the surface charge of the pore reducing co-ion exclusion and increasing event rates. At low salt concentrations, surface charge dominates contributing significantly to the total ion current. Conversely, at higher salt concentrations pore conductance is comparable to that of the bulk solution.^40^ Charge screening of analyte surface charge diminishes Δ*I* by adding counterions to the ion flux reducing the number of net displaced ions (*i.e.*, volume exclusion effect). Sufficiently enhanced analyte conductance, facilitated by a counterion cloud, can lead to observable conductive pulses.^40–42^

In sensors exhibiting concentration polarization, biphasic pulses may occur.^43,49^ In such cases, analyte migration through ion-rich regions results in resistive pulses, because current change is primarily due to volume exclusion of ions. Migration through ion-depleted regions produces conductive pulses when screening counterions sufficiently counteract the volume exclusion effect.^43,44^ For applications in which controlling surface charge is crucial, surfaces can be engineered to have the desired charge density *via* material selection, surface activation or modification, and/or buffer optimization.

## RPS signal, noise, and bandwidth characteristics

**Fig. 3:**
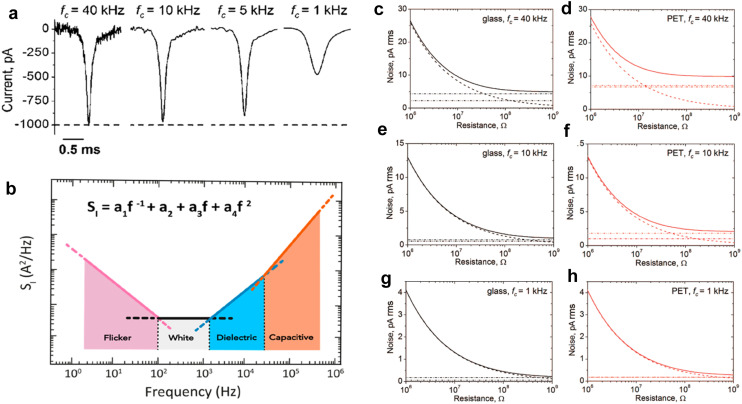
(a) Comparison of four current transient amplitudes obtained *via* electrokinetic translocation of icosahedral virus particle through conical nanopore collected at four different BWs. (b) Four different noise associated with RPS and their characteristic frequency response range. (c–h) Theoretical illustration of four different noise: (-----) thermal noise, (-·-··) headstage and amplifier noise, (-··-) dielectric noise, (⋯⋯) quantization noise, ( <svg xmlns="http://www.w3.org/2000/svg" version="1.0" width="32.333333pt" height="16.000000pt" viewBox="0 0 32.333333 16.000000" preserveAspectRatio="xMidYMid meet"><metadata> Created by potrace 1.16, written by Peter Selinger 2001-2019 </metadata><g transform="translate(1.000000,15.000000) scale(0.014583,-0.014583)" fill="currentColor" stroke="none"><path d="M80 480 l0 -160 960 0 960 0 0 160 0 160 -960 0 -960 0 0 -160z"/></g></svg> ) total noise generated by the glass and PET substrates at 1, 10 and 40 kHz frequencies. (a) and (c–h) Reproduced with permission from Uram *et al.*,^54^ Copyright 2008 American Chemical Society. (b) Reproduced with permission from Fragasso *et al.*,^55^ Copyright 2020 American Chemical Society.

In RPS, changes in ionic current due to a translocation event are transduced by the appropriate electronics. Application of a bias voltage with Ag/AgCl electrodes (reversible and non-polarizable)^50^ across an electrolyte solution on either side of the nanopore generates a baseline ionic current (*I*_0_), typically in the nanoampere (nA) range, with transient current changes as small as picoamperes (pA).^18,23,51–53^ Current amplifier circuity is able to amplify these changes to a measurable level and convert the RPS events to a voltage signal. High-frequency noise is then removed from the signal using a low-pass filter (LPF), which, in conjunction with the electronic amplifier circuitry and the RC time constant of the nanopore, establishes the system bandwidth (BW). A common challenge in RPS is that the timescale of translocation events (as short as tens of microseconds) can be shorter than the system's temporal resolution (temporal resolution ∼1/BW). A higher BW improves temporal resolution but also increases system noise, creating a trade-off in signal fidelity (see Fig. 3a and below for further discussion of BW).^54–57^

### Noise and BW

Noise and BW are essential parameters to consider in any RPS measurement. Complete resolution of an RPS event requires a specific BW. Operating at a low BW can lead to signal attenuation (*i.e.*, smaller amplitude) and pulse broadening (*i.e.*, wider pulse width),^15,54,58^ as illustrated in Fig. 3a; the signal amplitude is comparable for a system BW of 40 and 10 kHz, but the current trace at 40 kHz has higher system noise. It is standard practice to set a detection threshold based on the root-mean-square (RMS) noise in *I*_0_. Higher RMS noise necessitates a higher detection threshold, potentially excluding low-amplitude translocation events and lowering the overall event rate. Understanding the sources and characteristics of noise can aid in noise reduction in current traces. Increasing the BW improves the system's temporal resolution but also may permit higher-frequency noise to pass reducing the SNR.

Each component in the measurement system (*i.e.*, nanopore, headstage, amplifier, LPF, and digitizer) has a BW and the component with the lowest BW typically sets the upper limit on the operating temporal resolution.^54^ Nanopore substrates are typically dielectric materials (*i.e.*, SiO_2_, SiN_*x*_, glass, polymer), which have both resistive and capacitive components.^59^ The BW can be estimated from;^54,59,60^6
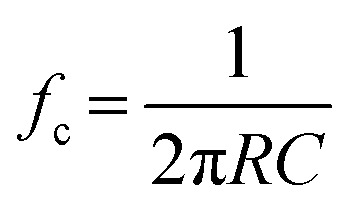
where *f*_c_ is the BW, *C* is the capacitance of substrate or membrane, and *R* (Ω) is the total resistance from the substrate. The combined BW of the system can be determined from the rise time (*T*_rise_) of the RPS event, which is the time it takes for a signal to rise from 10% to 90% of its amplitude;^54,61^7
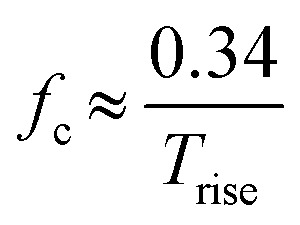
The type of noise is typically frequency-dependent (Fig. 3b).^55–57^ The noise can arise from various sources including environmental, amplifier electronics, and the pore itself. At low frequencies, also known as flicker or 1/*f* noise regime, the exact origin of this noise is unclear, but theories suggest it arises from surface effects causing fluctuations in the number of charge carriers (number fluctuation theory) and/or from fluctuations in their mobility (mobility fluctuation, or Hooge, theory).^56,62^ Sources that may also contribute to 1/*f* noise include protonation/deprotonation of surface groups within the nanopore, nanobubbles, mechanical instabilities in the measurement system, or surface adsorption/desorption characteristics within the nanopore. The presence of nanobubbles can intermittently alter the ion flow, and mechanical instabilities are potential cause for low-frequency baseline shifts. Proper surface engineering of nanopores, such as functional group modification or coating with low-noise materials, can mitigate issues associated with 1/*f* noise.

A nanopore behaves as a resistor within a sensor network and noise arising from a resistive component is thermal noise (also known as Johnson–Nyquist noise). Thermal noise results from the agitation of charge carriers inside the sensor.^63^ This type of thermal noise is white, meaning its power density is independent of frequency.^55,64^ Another component of white noise is shot noise and is due to the quantized nature of charge and arises from charge carriers crossing a potential barrier^65^ (*i.e.*, interface between electrode and buffer). White noise typically dominants at higher frequencies (1 kHz to 1 kHz or more) compared to 1/*f* noise.

At high frequencies (>1 kHz), dielectric materials used to fabricate the nanopore exhibit energy dissipation in the form of thermal energy contributing to a specific type of noise known as dielectric noise. This noise arises as a consequence of the insulating material not being able to perfectly respond to the rapidly alternating electric fields. Additionally, the capacitance of the substrate material can also generate capacitive noise, especially in high-impedance environments.^55^

An analog signal is converted to a digital signal using an A/D converter. Quantization is the mapping of the continuous values of an analog signal to discrete values representing the digital signal by the A/D converter. Each value to the nearest integer multiple of the A/D conversion is the smallest change in signal that the digitizer can distinguish.^63^ This approximation introduces a type of error known as quantization noise, which is the difference between the actual analog signal and the digitized signal. The conversion resolution is dependent on the bit depth of the digitizer. The higher the bit depth the smaller the quantizing step (also called the least significant bit, or LSB) and thus, lower quantization noise. In addition to quantization noise, the headstage and amplifier also can generate high-frequency capacitive noise (>10 kHz) from electrode wiring and coaxial cables, amplifier input, and feedback elements.^63,66^

Uram *et al.*^54^ determined the theoretical noise levels in a nanopore measurement and compared them to experimentally measured noise using an Axopatch 200B amplifier at 1 kHz, 10 kHz, and 40 kHz for glass, and polyethylene terephthalate (PET) substrates. While theoretical quantization noise was minimal across all conditions likely due to high digitizer resolution and system filtering. Thermal noise dominated at lower pore resistances (≤30 MΩ) due to increased current levels. At a BW of 10 kHz, thermal noise dominated up to resistances ∼400 MΩ (Fig. 3c–h). PET exhibited higher dielectric noise compared to glass, which was attributed to PET's higher capacitance and dissipation factors. At higher applied voltages, noise increased significantly and was attributed to 1/*f* noise. Patil *et al.*^67^ used molecular dynamic simulations to predict noise in solid-state nanopores (SSNPs) concluding that low-frequency (<100 Hz) 1/*f* noise was due to surface charge density aligning with the number fluctuation theory. Due to these noise sources, conventional patch-clamp amplifiers are limited to a BW of ∼100 kHz. However, most experiments are performed at ∼10 kHz to reduce overall noise levels and ensure signal fidelity.

### Noise suppression and temporal resolution for RPS measurements

In addition to employing appropriate high-pass filters (HPF) and LPF, several strategies can be incorporated to minimize noise in RPS measurements: (i) increasing pore resistance by reducing pore diameter or increasing pore length to reduce thermal noise; (ii) increasing surface hydrophilicity to reduce nanobubble formation^54^ or increase surface charge density leading to surface conduction dominating over bulk conduction all of which reduce flicker noise;^67^ (iii) selecting substrate materials with low dielectric loss to lower dielectric noise; (iv) use of extremely small area capacitor plates and shortening the wiring length between the amplifier and sensor to reduce capacitive noise; and (v) shielding the system with a Faraday cage to help isolate the RPS experiment from external electromagnetic interferences.

Digital post processing with bandpass filters are routinary used to reduce the RMS in *I*_0_.^68,69^ Bessel filters are commonly used as LPF, but can also attenuate blockade signal amplitude especially for fast translocation events. Even in the case for events that are scored properly, high-frequency components in the RPS signal can be lost as well when employing LBF.^70^

Wavelet and deep learning have also been proposed to reduce noise for RPS measurements. For the wavelet method, the signal and background noise are separated in the wavelet domain with a breakdown of the signal that shows the time and frequency of the events allowing large-magnitude wavelet components to remain, while small-magnitude components are eliminated.^69^ In the case of deep learning, the Noise-2-Noise technique uses pairs of noisy nanopore signals to denoise them without requiring a clean signal and then employing convolutional neural networks to learn the mapping from noisy data followed by a deconvolution or reconstruction of the data revealing features in the signals that otherwise would be hidden.^71^

Rosenstein *et al.*^66^ developed a custom-built preamplifier using complementary metal-oxide semiconductor (CMOS) technology that was tested by integrating it to a SSNP embedded within a thin SiN_*x*_ membrane. The amplifier could operate at a BW of 1 MHz and produced a SNR >5 for RPS events at a temporal resolution of 1 μs. The preamplifier circuitry, positioned directly within the fluid chamber, reduced parasitic capacitance from the SiN_*x*_ and electrode (Ag/AgCl electrodes fabricated directly on the preamplifier surface), thereby lowering high-frequency capacitance noise. Shekar *et al.*^72^ later implemented a similar CMOS setup with a 10 MHz BW that was able to achieve a temporal resolution approaching 100 ns with a SNR of 10 at 5 MHz BW. Hartel *et al.*^73^ utilized a 500 kHz BW with a temporal resolution of 2 μs to perform a beta distribution analysis to observe events as fast as 35 ns. For a more extensive discussion on high BW approaches in nanopore recordings, readers are directed to this comprehensive review.^74^

Nanopore sensors can be modeled as a resistor–capacitor (RC) circuit that will determine the speed of the ionic current modulation measurements. In this context, in-plane pores fabricated in a polymer or glass exhibit distinct RC characteristics due to their unique material properties, pore size, geometry, and surface chemistry. Combined with the polymer capacitance and its unique dielectric properties, this will result in characteristic resistance–capacitance filtering.^75^ Conversely, out-of-plane pores create a RC circuit resulting in high-capacitance limiting time resolution.^55^

### Sampling rate and signal aliasing in RPS

Sampling rate (Hz) is the number of data points recorded per unit time and the appropriate rate is dependent on the type of analysis to be performed. For time-domain analyses (*e.g.*, pulse analysis), sufficient measurements must be recorded to ensure sampling intervals are shorter than translocation events to prevent signal distortion or missed events. According to the Nyquist theorem,^76^ the sampling frequency should be ≥2× the system BW. However, in nanopore RPS applications a rate of ≥10× the BW is often used.^9,15,21,24^ For frequency-domain analyses (*e.g.*, noise analysis), it is essential to prevent aliasing, a phenomenon where high frequency components (above the Nyquist frequency) fold back into the lower frequency range distorting signals in the frequency range of interest. To prevent this, a low-pass anti-aliasing filter (a filter with a sharp attenuation) can be placed before signal digitization. Butterworth and elliptic filters are commonly used in frequency-domain analyses due to their sharp attenuation. In contrast, Bessel filters are common for time-domain analyses due to their lower overshoot, a phenomenon where the recorded signal exceeds the expected value during a transient response.^63^ Oversampling is less common in frequency-domain measurements not because it causes aliasing, but because it may unnecessarily increase data volume without any improvement in frequency information.

In the sections that follow, different pore configurations (in-plane *versus* out-of-plane) and the use of different materials for nanopore measurements will be discussed. Regardless of the pore configuration or material, noise contributions to the RPS data remain relevant as discussed above, even though their magnitude and impact may vary depending on the pore configuration and/or material used for the nanopore. Also, the underlying physics of RPS signal generation are similar in all cases. We will highlight these common principles and variations as we explore each of the pore materials and configurations discussed in the following sections.

## Pore configurations: out-of-plane *vs.* in-plane pores

Accurate detection and identification of analytes are not only determined by the driving voltage, buffer composition, and electronic parameters (see eqn (4) and (5)), but also the associated nanopore shape and configuration. While the pore size is important in terms of determining the SNR in any RPS measurement, the pore configuration including its orientation with respect to the translocation direction (out-of-plane *vs.* in-plane), surface chemistry, and shape can have a profound impact on the measured response as well as noise contributions to the measurement. For example, adjusting pore geometry can enhance the detection accuracy and sensitivity.^11,77^ Additionally, modifying surface properties such as charge density or functional groups^11,23,78^ can affect potential analyte–nanopore wall interactions, concentration polarization and/or ICR.

Nanofluidic sensors in general contain micro- and nanoscale structure interfaces. Such interfaces may introduce entropic barriers during the voltage-driven translocations of large biomolecules, that are not observed in microscale structures.^8,33,79–82^ Proper configuration of the micro-nanostructure interface can lower the entropic barrier providing smooth entry of macro-molecules into the nanopore, thereby increasing event capture rate.^83^ Additionally, surface charge effects of the pore must be carefully considered as they can determine the amount of concentration polarization, ICR, and/or translocation time and signal amplitude, all of which affects overall sensor performance.

Nanopore geometries broadly speaking can consist of the out-of-plane^11,12,21,84^ or the in-plane configuration.^24,85^ In this review, we define out-of-plane nanopore sensors as those in which the analyte translocates through the pore orthogonally to the sensor's substrate, while for in-plane pores the analyte translocates parallel to the sensor substrate. In-plane and in some cases, out-of-plane nanopores can be designed as a single pore, multiple pores in series,^51,86^ or multiple pores in parallel,^17^ which can increase the information content of nanopore RPS measurements (serial configurations) or the measurement throughput (parallel configurations), respectively.

## Out-of-plane nanopores

### Out-of-plane biological nanopores

Over the past few decades, many researchers have widely used biological out-of-plane nanopores in a broad range of applications including DNA/RNA sequencing, and protein analysis to name a few. They can be engineered by various techniques preserving their physical and chemical stability across different devices.^87^ Examples of different biological nanopores includes α-HL,^88,89^ MspA,^87,90^ and *φ*29.^91^Table 1 summarizes their important operational parameters. The α-HL nanopore from *Staphylococcus aureus* possesses a molecular mass of ∼33 kDa and contains three distinct regions; the *cis* entrance, central constriction, and *trans* exit.^88,92^ The α-HL pore has been utilized for DNA sequencing as it allows the translocation of single stranded (ss) DNA through the nanopore with each nucleotide generating distinct RPS signals due to their structural variations (Table 1a).^88^ The MspA porin nanopore, on the other hand, is extracted from the *Mycobacterium chelone* and contains a more cylindrical shape (Table 1b).^90^ Its octameric structure can make it an ideal candidate for nanopore sequencing because of the ability to generate distinctive RPS signals for A, T, C and G.^93^ The third nanopore type is *φ*29 from *Bacillus subtilis* and has a propeller-like shape (Table 1c).^91^

**Table 1:** Biological nanopores. Structural images and pore diameters of (A) α-HL, (B) MspA, and (C) *φ*29 from side (left) and top (right)

	Nanopore type	Structural view	Pore diameter	References
a	α-HL	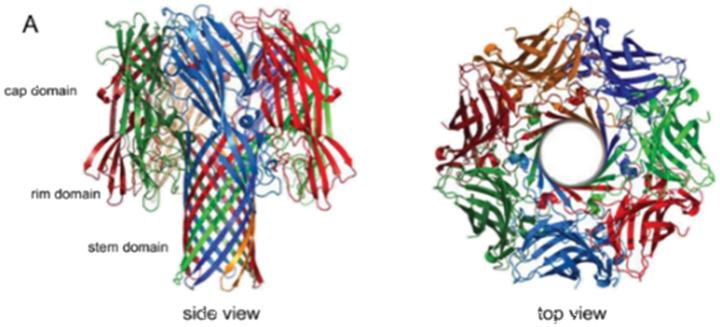	1.4 nm	Tanaka *et al.*^88^
				Ying *et al.*^89^
b	MspA	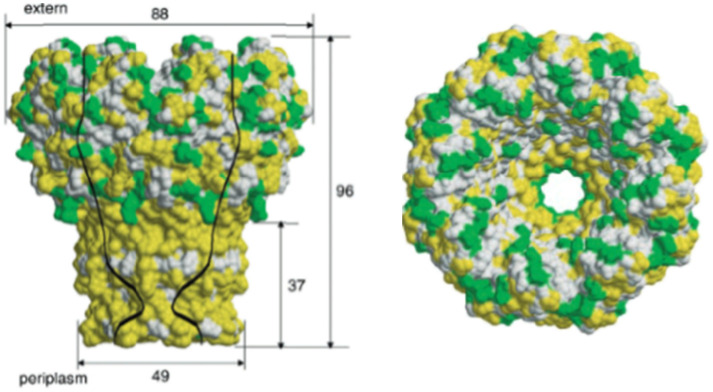	1.2 nm	Faller *et al.*^90^
c	*φ*29	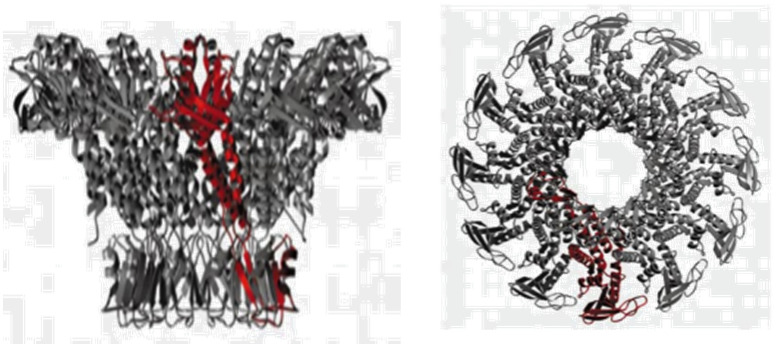	3.5 nm	Guasch *et al.*^91^

a Reproduced with permission from Tanaka *et al.*,^88^ Copyright 2011 The Protein Society.

b Reproduced from Faller *et al.*^90^ with permission from American Association for the Advancement of Science.

c Reproduced with permission from Guasch *et al.*,^91^ Copyright 2002 Academic Press.

Stoddart *et al.* compared the RPS behavior of wild-type (WT) and E11N/K147N modified α-HL nanopores by shuttling homopolymer and heteropolymer ssDNAs through the pores and observing current drop amplitudes.^94^ They immobilized oligonucleotides at the pore vicinity *via* biotin tags at their 3′ termini. Upon application of a bias voltage, the oligonucleotides were captured by the nanopore and held there over a period of time, while residual current generated by each nucleotide were detected and recorded.

**Fig. 4:**
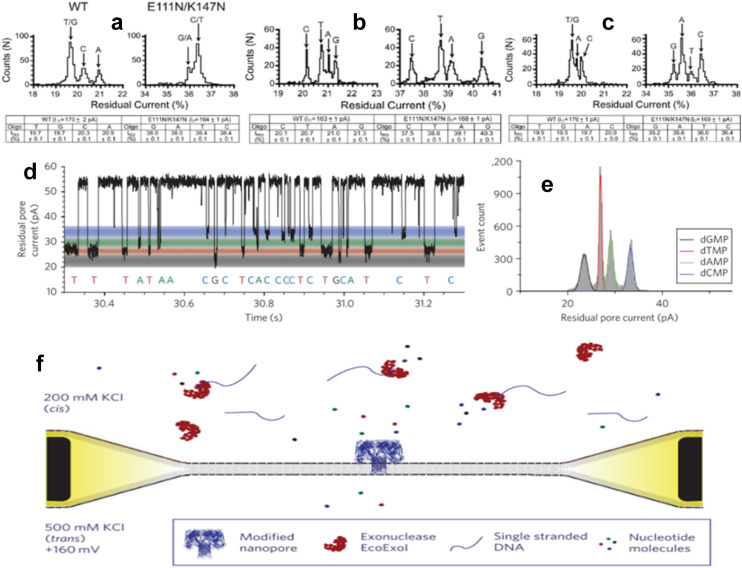
Molecule translocation through biological nanopores. Histogram plot (top) for *I*_res_ and a table (bottom) highlighting the average *I*_res_ for DNA/α-HL pore interaction where oligonucleotides have a sequence of (a) 5′-C_31_NC_7_Btn-3′, (b) 5′C_26_N_13_Btn-3′, and (c) 5′C_22_N_17_Btn-3′. Reproduced from Stoddart *et al.*^94^ with permission from Proceedings of the National Academy of Sciences. (d) Current trace generated by translocation of dNMPs through modified α-HL nanopore and (e) Gaussian fit histogram plot of *I*_res_ for in the presence of four nucleotides. (f) Schematic image of ssDNA/Exo-1 interaction in the *cis* chamber and produced mononucleotide translocation through modified α-HL nanopore. Reproduced from Clarke *et al.*^95^ with permission from Proceedings of Nature Nanotechnology.

**Fig. 5:**
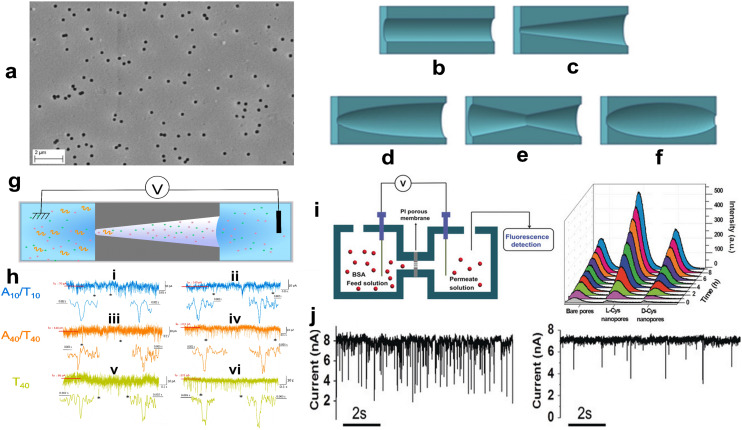
(a) SEM image of a multi-porous (10^8^ nanopores per cm^2^) PET membrane etched for 120 min (*d*_base_ 232 nm). Nanopore geometries: (b) cylindrical, (c) conical, (d) bullet-like, (e) double-conical (hourglass), (f) cigar-shaped track etched nanopores. Reproduced from Kaya *et al.*^139^ with permission from The Electrochemical Society. (g) Experimental setup for DNA (A10/T10, A40/T40 and T40) sensing through track-etched PET conical nanopore. (h) Examples of current traces and zoomed-in current blockade traces recorded for A10/T10 at (i) 250 mV and (ii) 500 mV; A40/T40 at (iii) 250 mV and (iv) 500 mV; T40 at (v) 250 mV and (vi) 500 mV. The symbol * identifies examples of current blockade signals zoomed below each trace. The current traces were obtained using pore with tip diameter = 3 nm and base diameter = 200 nm. Reproduced from Meyer *et al.*^143^ with permission from the Multidisciplinary Digital Publishing Institute. (i) Schematic illustration of BSA transport through a chiral porous membrane (i). The driving voltage was +2 V (anode was in the permeate side). (ii) Time-dependent fluorescence change when BSA was allowed to be transported through bare nanopores and l/d-Cys immobilized nanopores. (j) Time-dependent current changes during BSA translocation in l- (i) and d-Cys (ii) modified single nanopores under the same conditions. Reproduced with permission from Zhang *et al.*,^142^ Copyright 2017 Wiley-VCH Verlag GmbH & Co. KGaA, Weinheim.

The specific nucleotide sequences used in these experiments consisted of 5′CCCCCCCCCCCCCCCCCCCCCCCCCCCCCCCNCCCCCCCCBtn-3′ (Fig. 4a), 5′CCCCCCCCCCCCCCCCCCCCCCCCCCNCCCCCCCCCCCCCBtn-3′ (Fig. 4b) and 5′CCCCCCCCCCCCCCCCCCCCCCNCCCCCCCCCCCCCCCCCBtn-3′ (Fig. 4c), where N was either G, A, T, or C. Both nanopores were used to distinguish the nucleotide at the “N” position. The E11N/K147N modified α-HL nanopore provided superior performance on the identification of nucleotide-specific current levels over its WT counterpart by comparing their *I*_res_ (residual current) distribution. Clarke *et al.* mutated α-HL nanopore to contain an am_6_amPDP_1_βCD adapter with a cysteine substitution at position 119, 121, 123, 135 or 137 amino acid residues. Electrokinetic translocation of single dNMP molecules through the nanopore produced the current trace shown in Fig. 4d.^95^ The modified nanopore established discrimination of all four nucleotides at the 99% confidence level (Fig. 4e). They further investigated the RPS performance of this mutated nanopore by translocating ssDNA mixed with *Exo*-1 and detected RPS signals produced by the enzyme-assisted DNA digestion of DNA under asymmetric salt conditions (see Fig. 4f).^95^

While biological nanopores are known for their exceptionally small size and relatively low noise, there are several challenges associated with them.^87^ The process of designing, fabrication, and modification of biological nanopores are often time-consuming and complex. Additionally, laboratory-purified proteins are available in limited quantities, which makes large-scale nanopore analyses difficult.^87^ Also, the fragility of the lipid bilayer can be problematic, especially when an electric field is applied across the nanopore limiting its operational lifespan.^96^ However, efforts have been made to stabilize the supporting biological membrane such as encapsulation of free-standing planar lipid membranes in a polymer hydrogel *via in situ* photopolymerization of poly(ethylene glycol)dimethacrylate (PEGDMA).^97^

### Out-of-plane solid-state nanopores (SSNPs)

Out-of-plane SSNPs are typically fabricated in free-standing membranes such as silicon nitride (SiN_*x*_), silicon dioxide (SiO_2_),^98^ glass,^99^ or ultrathin 2D materials such as molybdenum disulfide^100^ or boron nitride.^101^ These membranes are placed between two fluidic reservoirs made out of materials such as Teflon,^21^ poly(dimethylsiloxane),^99^ polymethylmethacrylate,^102^ or polycarbonate.^103^ The membrane-embedded out-of-plane pores can be fabricated using transmission electron microscopy (TEM),^104^ where an electron beam is tightly focused to a desired spot on the membrane and used to open the membrane to crease a nanopore. While TEM offers high precision, it requires expensive instrumentation and has low throughput; typically producing ∼1 device per hour. Focused ion beam (FIB) milling using Ga^+^ (ref. 105 and 106) or He^+^ ions^107^ improves device production throughput allowing the fabrication of 20–30 devices per hour. Controlled dielectric breakdown (CDB)^108,109^ has emerged recently wherein an applied voltage is used to form a pore at a membrane defect. This approach does not require sophisticated equipment and in principle, allows for parallelized fabrication to increase production. However, CDB has limitations. For example, pore formation in CDB is stochastic, leading to variability in pore size and location. It is also limited to producing single-pore devices and thus, challenges associated with increasing parallelization. Moreover, the nanopores created by CDB tend to grow over time limiting their long-term stability and utility.

### Inorganic membrane pores

Table 2 summarizes various membrane-based nanopore types. The pore developing process within the membrane requires precise alignment of the ion beam on the membrane surface and controlling the exposure time to provide the most desirable pore shape and diameter. When the dosage of the pore-forming beam is carefully controlled, the size of a nanopore can be adjusted to <5 nm.^110^

**Table 2:** Membrane based SSNPs. Schematic view (left) and SEM/AFM images (right) of SiN_*x*_ (a and b), MoS_2_ (c and d), graphene nanoribbon (GNR) (e)

Pore type	Pore height	Pore diameter	Pore fabrication	Schematic view of experimental set up (left) and SEM/AFM images of nanopore size	Reference
aSiN_*x*_	>20 nm	>4.8 nm	HF etching	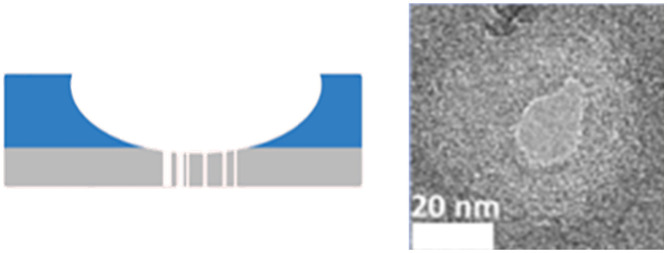	Xia *et al.*^119^
bSiN_*x*_	∼10 nm	<1 nm	Electron bean-induced sputtering	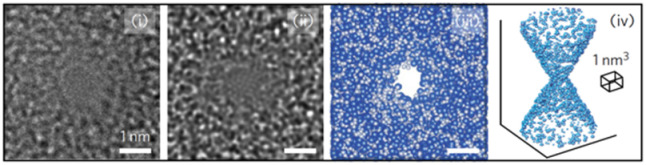	Kennedy *et al.*^121^
cMoS_2_/SiN_*x*_	0.7 nm (MoS_2_)/20 nm (SiN_*x*_)	>5 nm	TEM	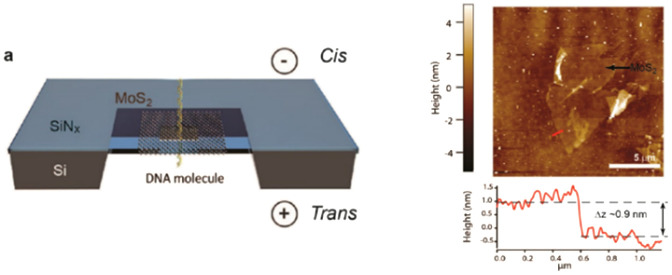	Liu *et al.*^100^
dMoS_2_	∼10 nm	∼0.8 nm	CVD	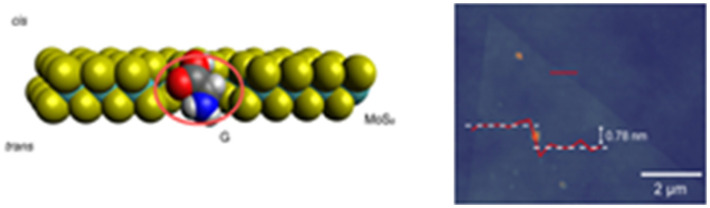	Wang *et al.*^127^
eSiN_*x*_/GNR	0.335 nm (GNR)/20 nm (SiN_*x*_)	∼10 nm	TEM	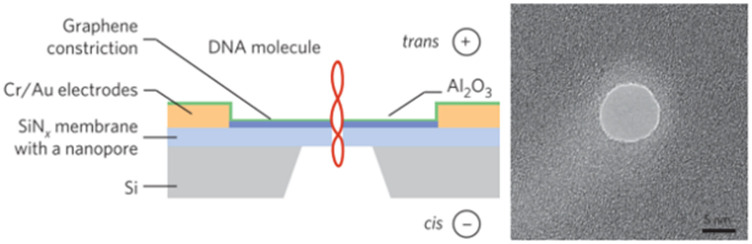	Traversi *et al.*^126^

a Reproduced with permission from Xia *et al.*,^119^ Copyright 2022 American Chemical Society.

b Reproduced from Kennedy *et al.*^121^ with permission from Proceedings of Nature Nanotechnology.

c Reproduced from Liu *et al.*,^100^ with permission from American Chemical Society.

d Reproduced from Wang *et al.*^127^ with permission from Proceedings of Nature Nanotechnology.

e Reproduced from Traversi *et al.*^126^ with permission from Proceedings of Nature Nanotechnology.

Membrane-based nanopore fabrication and implementation was first reported by Li J. *et al.* in 2001,^111^ who used an argon (Ar^+^) ion beam to generate 5 nm pores on the surface of a bowl-shaped SiN_*x*_ membrane.^111^ When testing the nanopore, a constant ionic current (*I*_0_) of 1.66 nA resulted at 120 mV applied voltage. When a 500 bp dsDNA was electrokinetically translocated through the nanopore, the results indicated current blockage dwell times in the millisecond regime. Current reductions (Δ*I*) were 88% lower compared to the open-pore current value.^111^

### Silicon nitride nanopores

SiN_*x*_ membranes are made of materials that exhibit excellent mechanical and chemical stability. Particularly, the nanopore size and shape are more adjustable in comparison to biological nanopores.^112^ The fabrication process of the pores within the SiN_*x*_ membrane window involves: (1) deposition of SiN_*x*_ film on the Si substrate; (2) photolithography and reactive ion etching to open a window on the membrane; and (3) nanopore formation using FIB milling,^111,113^ electron beam lithography (TEM),^114,115^ or laser assisted drilling.^116,117^ Alternatively, chemical etching methods can be employed for nanopore formation.^118,119^ Xia *et al.* fabricated 20–100 nm thick low-stress SiN_*x*_ membranes on a glass substrate (Table 2a and b).^119^ Then, nanopores with diameters ranging from sub-nm to 100 nm were chemically etched into the membrane using hydrofluoric acid (HF).^119^ Zvuloni *et al.* employed laser drilling (LD) to fabricate pores in the 2.5–5 nm range.^116^ These nanopores were subsequently used for analyzing single protein molecules. Unlike biological nanopores, SiN_*x*_ can be integrated with other techniques to enhance signal sensitivity. For example, Dela Torre *et al.* integrated TiO_2_ coated SiN_*x*_ nanopore arrays (6 × 6) with total internal reflection fluorescence (TIRF) spectroscopy to analyze 1 kbp dsDNAs, which were covalently attached to streptavidin/quantum dots.^120^ Non-propagating evanescence radiation was able to excite the fluorophores only when the DNA-dot duplexes were electrokinetically directed towards the nanopore offering a unique and highly selective DNA detection technique.^120^ Timp's group^121^ reported a sub-nanometer SiN_*x*_ pore for protein sequencing, which used a quadromer (four amino acid residue) appendage to resolve single residues with volume differences of ∼0.07 nm^3^.

### Aluminum oxide nanopores

Aluminum oxide (Al_2_O_3_) nanopores are gaining interest due to their optical and electrochemical properties, pore size adjustability, and high thermal stability.^122^ Compared to SiN_*x*_-based nanopores, Al_2_O_3_ nanopores offer lower noise, which is beneficial for high-sensitivity detection.^123^ A study by Venkatesan *et al.* demonstrated that DNA translocation was significantly slower using an Al_2_O_3_ nanopore compared to SiN_*x*_ nanopores.^124^ The primary reason for this observation was ascribed to electrostatic attraction between the negatively charged DNA molecules and the positively charged Al_2_O_3_ nanopore walls, which partially reduced the speed of DNA during translocation.^124^

### Two-dimensional nanopores

Monolayer or 2D nanopores are exceptionally thin, which result in increased RPS signal amplitudes, but consequently short dwell times, which makes the system BW considerations critical. Commonly used materials for this platform are MoS_2_ or graphene.^125^ Liu *et al.* fabricated MoS_2_ a nanopore sensor with 0.7 nm thickness and compared its performance to SiN_*x*_ by translocating λ-DNA through the 2D nanopores (Table 2c and d).^100^ The current transient data revealed a 5-fold increase in signal amplitude and a 10-fold improvement in SNR.^100^ Traversi *et al.* was able to deposit a 0.335 nm thin graphene layer on a SiN_*x*_ membrane surface by chemical vapor deposition (CVD) (Table 2e).^126^ They integrated graphene nanoribbons (GNR) across the membrane and electrokinetically translocated DNA molecules through the nanopore employing two detection modes; current-based and electrical-based modes. The current-based mode was initiated by RPS criteria, and the electrical-based mode was achieved *via* inducing the transmembrane potential perpendicular to the DNA translocation direction.^126^

The 2D pores have recently been demonstrated to detect individual amino acids, post-translational modifications,^127^ single nucleotides^128^ and DNA molecules.^100^ 2D nanopores have also been used in studies of neurodegenerative disease with results associated with the characterization of oligomers of amyloid-forming proteins demonstrating high resolution when compared to a similar characterization using TEM or mass photometry.^129^

There are challenges with 2D nanopores such as short dwell times, which often exceeds the operational BW of the measurement system.^112^ Also, there are challenges associated with membrane current leakage introduced during film growth and membrane fabrication, damage of the 2D material due to electrical discharges, and the fact that the thin membrane nanopores are prone to widening and damage when in solution. These and other challenges as well as some alternative solutions are discussed in detail by Graf *et al.*^130^

Another important consideration is that because the ionic current through a nanopore is inversely proportional to its length and the translocation of a molecule blocks a large fraction of the ions occupying the pore volume, 2D nanopores have the advantage of generating high Δ*I* values.^130^ However, the thin MoS_2_ membranes come with a consequence of low mechanical stability as well as charge fluctuation of its suspended membrane, which increases 1/*f* noise. Thus, the signal to noise ratio (SNR) is ∼10,^131,132^ and consequently not so different from the values observed in pores constructed in SiN_*x*_ membranes.^133^ Another challenge with graphene nanopores arises from clogging caused by hydrophobic interactions between DNA and graphene requiring a coating such as a self-assembled pyrenediol monolayer.^134^

Another interesting membrane-based out-of-plane pore system is MXene consisting of early transition metal carbides and/or nitrides of chemical formula M_*n*+1_X_*n*_T_*x*_, where M is an early transition metal, *x* denotes carbon and/or nitrogen, and T_*x*_ represents surface functional groups such as –O–, –OH, –F, or –Cl.^135^ Mojtabavi *et al.*^136^ reported the development of a liquid–liquid interface assembly of Ti_2_CT_*x*_ and Ti_3_C_2_T_*x*_ MXene sheets with an ∼60% process yield rate for producing the nanometer-sized pores in free-standing MXene membranes. The MXene pores exhibited lower noise characteristics when compared to the other 2D systems, such as graphene or MoS_2_. However, the MXene pores show challenges as do other 2D membrane pores such as poor structural stability, oxidation, and high-cost production.^135,137^

### Track etched nanopores

Track etched nanopores are a class of out-of-plane SSNP fabricated by ion irradiation followed by chemical etching of polymer membranes, such as polycarbonate (PC) or polyethylene terephthalate (PET) (Fig. 5). This fabrication strategy enables production of cylindrical or conical shaped pores with tunable dimensions.^138,139^ In the standard fabrication process, high energy heavy ions (*e.g.*, Kr, Xe, Au) are accelerated and bombarded onto a polymer film creating latent tracks, which form along the path the heavy ions travel. Subsequent chemical etching, usually done with a strong base such as NaOH, selectively removes damaged regions, which then form nanopores with size and shape that can be tuned by adjusting the etching time, temperature, and chemical concentration.^140^

Track-etched nanopores can be made into various shapes as shown in Fig. 5a–e. The shape of the pore directly affects the electrical signal, translocation speed, and rectification behavior of ions and molecules passing through them. Cylindrically shaped pores (Fig. 5b) have a constant diameter throughout the pore length and are formed by symmetric etching from both sides of the membrane. These pores are commonly used for size-based sensing and transport studies of single molecules. Conically shaped pores (Fig. 5c) have a narrow tip at one end of and a wider base at the opposite end and are generated by asymmetric etching (etching one side more aggressively than the other). These pores exhibit current rectification and are useful for directional transport and gating applications. The double conically shaped pores (Fig. 5d) provide symmetrical electric fields and can enhance analyte capture rate. These pores are created by etching both sides of the membrane in a controlled manner to form two conical shapes that meet at the center of the membrane. Bullet-like track-etched pores (Fig. 5e) refer to a pore geometry that combines a steep taper near the tip with a gradual widening toward the base resembling the shape of a bullet. During the fabrication, one side of the membrane is etched more aggressively often using a high-concentration of NaOH and etching is stopped quickly after breakthrough limiting enlargement of the tip. These pores have the ability for electric field focusing, translocation control, and improved directional selectivity. The cigar shaped pores (Fig. 5f) are a variant of asymmetric conical pores, where the pore gradually tapers from a wider base at the middle to a narrower tip at both ends and resembles the rounded profile of a cigar. This shape is fabricated through controlled etching. The gradual tapering of the shape produces a spatially varying electric field, which can help slow down the translocation of biomolecules like DNA or proteins. The smoother geometry also reduces fluctuations in the ionic current, which leads to increased SNR of RPS signals.

As an alternative approach for producing conical pores in a plastic, Choi *et al.* utilized UV nanoimprint lithography (UV-NIL) of a SU-8 resin to produce an array of pores.^141^ In this process, the Si master consisted of an array of needles with a tip diameter of ∼25 nm. Following UV-NIL, the SU-8 membrane could be heated to near its glass transition temperature to induce polymer reflow to allow shrinkage of the pores at a rate of 2.7 nm min^−1^ that could produce polymer pores of <10 nm. The device was used to detect the translocation of λ-DNA through the pores.

A study done by Meyer *et al.* demonstrated the use of track-etched nanopore in PET membranes for ssDNA and dsDNA translocation as shown in Fig. 5f and g.^143^ Analysis using current blockade amplitudes and dwell times resulted in poor resolution due to overlapping signals among different DNA types (A10/T10, A40/T40 and T40). Machine learning significantly enhanced the discriminatory power of a conical track-etched nanopore for detecting short DNAs with a classification accuracy from ∼50% (using two features) to as high as 82% (using all five features), especially at lower voltages and smaller pore sizes (see Fig. 5h(i–vi)).

The ability to modify chemically the surface of track-etched nanopores is an advantage of these out-of-plane pores. Surface modification of the pore with charged or biological molecules can improve the selectivity of the measurement or control analyte translocation speed. For example, Thangaraj *et al.* demonstrated ssDNA (40 mer) and dsDNA (10 mer and 40 mer) translocation through PET track-etched nanopores coated with Al_2_O_3_ by atomic later deposition.^144^ Modified pores with Al_2_O_3_ (isoelectric point ∼5) generated dwell times of 0.85 ms, 1.07 ms, and 0.65 ms for ssDNA, 10 mer dsDNA and 40 mer, respectively.

In another example, Zhang *et al.* studied selective protein (BSA; bovine serum albumin) translocation through chiral-modified conical shaped nanopores fabricated in a polyimide (PI) membrane.^142^ The experimental setup is shown in Fig. 5i(i). When the BSA was labeled with a fluorescent dye, they could use fluorescence detection to monitor how much protein was able to translocate through the nanopore membrane; d-Cysteine modified pores provided higher efficiency transport through the pore compared to a bare pore and a pore containing l-cysteine (see Fig. 5i(ii)). l-Cysteine modified (l-Cys) nanopores improved the protein capture rate compared to pores modified with d-cysteine (12.9 events per s for l-Cys pores and 1.6 for d-Cys pores; see Fig. 5j(i) and (ii)). This phenomenon was caused by BSA drawn into the modified nanopores using chiral gate modulation.^142^

As a final example, Ma *et al.* investigated the translocation of hyaluronic acid, HA, through glass conical nanopores both with and without immobilized enzymes.^145^ Without enzymes, the HA transit through different pore diameters and polymer concentrations followed de Gennes' scaling laws. When hyaluronidase was grafted at the base side of the pore, HA chains were enzymatically cleaved at the entrance, and degradation products were detected downstream; grafting at the tip side led to prolonged dwell times, reflecting HA–enzyme binding interactions before degradation.

### Multi-pore sensors using in-series or in-parallel configurations

**Fig. 6:**
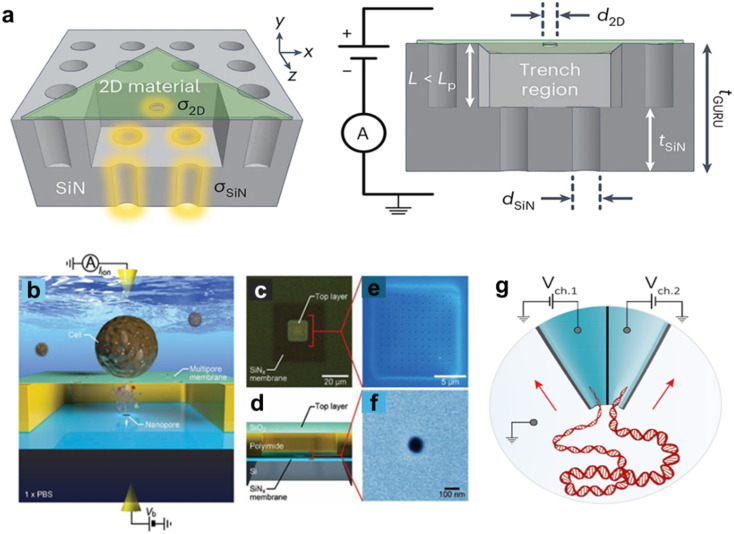
(a) Fabrication schematic involving electron beam lithography (EBL), etching, transfer of two-dimensional materials, and alternating current scanning transmission electron microscopy sculpting (AC-STEM). SiN nanopores serve as a guiding and reusable (GURU) platform. The pore thicknesses are denoted as *t*_SiN_ and *t*_2D_, the diameters as *d*_SiN_ and *d*_2D_, and the overall thickness is represented as *t*_GURU_. Device configurations are represented as [N, M], for NSiN pores and M 2D pores. *σ*_SiN_ and *σ*_2D_ represent the surface charge densities of silicon nitride (SiN) and 2D nanopores, respectively. Experimentally interlayer separation, L, can be manipulated by adjusting the time of reactive ion etching (RIE). Reproduced with permission from Chou *et al.*,^146^ Copyright with permission from Proceedings of Nature Nanotechnology. (b) A diagram showing a 3D architecture for single-cell lysis to DNA detection by ionic current measurement. A voltage, *V*_b_, applied across the vertically stacked two nanopore membranes and the corresponding ionic current measured monitored with a pair of Ag/AgCl electrodes in phosphate buffered saline (PBS). (c) A photographic view of the integrated nanopore emphasing the top multipore sheet. The 50 nm-thick SiN_*x*_ nanopore membrane in dark square area. (d) A 3D model illustrating the cross-sectional architecture of the integrated nanopore. (e and f) False-colored scanning electron micrographs of the SiO_2_ multipore and SiN_*x*_ nanopore. Reproduced with permission from Tsutsui *et al.*,^147^ Copyright 2021 Wiley-VCH GmbH. (g) Schematic depiction of the experimental configuration illustrating a twin barrel nanopore. Reproduced from Cadinu *et al.*,^148^ with permission from American Chemical Society.

Out-of-plane nanopores can be arranged with a dual in-series or parallel configuration. For the in-series configuration, a layer-by-layer fabrication strategy is typically employed. For example, Chou *et al.*^146^ reported the use of a bilayer-coupled in-series nanopore sensor consisting of a vertically stacked architecture The sensor contained a top two-dimensional (2D) layer with a single pore ∼2 nm in diameter and a bottom SiN_*x*_ layer with one or more pores ranging from 10–31 nm in diameter depending on the fabrication method (Fig. 6a). The dual-pore in-series sensor was used for the guiding, tracking, and detection of single molecules.

Tsutsui and coworkers described a 3D-integrated nanopore device for the detection of single DNA molecules.^147^ The device consisted of a SiO_2_ multi-pore sheet stacked vertically on a SiN_*x*_ nanopore membrane with all layers integrated onto a Si wafer. RPS was used to reveal signatures indicative of the folding states of polynucleotides. The authors demonstrated the device's ability to detect *E. coli*-derived DNA without amplification (Fig. 6b–f).

In a parallel dual out-of-plane configuration, Cadinu *et al.* reported a double-barrel nanopore design for regulating the molecular transport of single molecules.^148^ The two pores were ∼20 nm apart and located at the tip of a double-barrel quartz nanopipette (Fig. 6g).

## In-plane nanopores

A unique feature of in-plane nanopore sensors is in their architectural flexibility. In contrast to out-of-plane sensor configurations, where the nanopore lies within a membrane separating two vertically aligned electrolyte-filled reservoirs, in-plane sensors have nanostructures that connect input and output microscale channels parallel to the surface. These micro/nanochannel interfaces use either a direct interface (*i.e.*, blunt interface) or engineered structures to create a size-gradient (tapering geometry) of nanochannels that can enhance sampling efficiency. Also, unlike out-of-plane sensors, in-plane sensors enable simple integration with planner microfluidics facilitating sample pre-processing prior to the RPS measurement. In-plane nanopore sensors can also contain a single nanopore, multiple nanopores in series (to sequentially detect analyte properties), or arrays of parallel nanopores (to enhance throughput), depending on the measurement needs. The use of multiple nanopores enable unique measurements such as the time-of-flight (ToF) that are not achievable in single nanopore systems.

As shown in Fig. 3, the noise in any RPS measurement arises from various sources including 1/*f* noise, white noise, and high-frequency noise typically resulting from capacitive and dielectric characteristics of the sensor. Beyond noise in any nanopore measurement, another key factor must be considered is the mass detection limit, which is related to the sampling efficiency of the sensing system. Although nanopore sensors can detect single molecules, they suffer from poor sampling efficiency. As a result, large mass loads of sample are required for the sensor to achieve reasonable detection rates (*i.e.*, high capture rates). While increasing the applied voltage can increase the frequency of RPS events, it also increases the analyte velocity, which can detrimentally affect the event rate due to BW issues.

### Glass (inorganic) in-plane nanopores

In-plane nanopores can be fabricated in glass or quartz substrates *via* e-beam lithography^149^ or focused ion beam milling, which are considered top-down fabrication strategies.^150,151^ The attractive nature of this fabrication strategy is that multiple pores (≥2) can be placed in series to offer some unique measurement opportunities as discussed above. For example, each nanopore in-series serves as an independent sensing element allowing transduction of the analyte more than once. Also, as an analyte moves sequentially from one pore to the next, the analyte's *μ*_app_ can be calculated based on the distance between the two nanopores, the electric field strength, and the ToF between the pores. It should be noted that *μ*_app_ is not nanopore-dependent and this can produce an RPS-independent and molecular-dependent characteristic that can aid in molecule or nanoparticle identification.

**Fig. 7:**
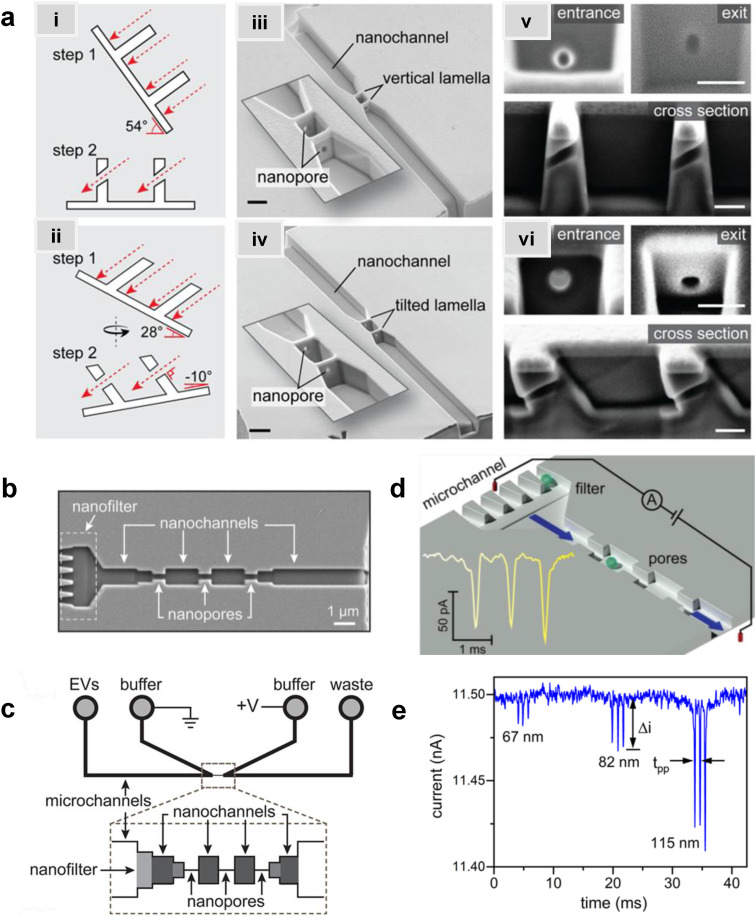
Glass in-plane nanopore devices. (a) Schematic view of vertical (i) and tilted lamella (ii) fabricated by adjusting the stage and the incident beams angles (ii), SEM images of vertical lamella (iii) and tilted lamella (iv) holding two in-plane circular nanopores directly milled on the lamella walls (scale bar: 1 μm). SEM images of conical (v) and cylindrical (vi) nanopore shapes initiated due to lamella wall alignments (scale bar: 200 nm). Reproduced from Zhang *et al.*^85^ with permission from American Chemical Society. (b) SEM image of three in-plane nanopores milled by FIB instrument. (c) Schematic diagram of the entire device containing 4 reservoirs, 2 V-shape microchannels, nanofilters, nanochannels and nanopores. (d) A visual illustration of the EV particles translocating through the nanopores and generating current transient amplitudes. (e) Current transient amplitudes of 67, 82 and 115 nm EV particles. Reproduced from Young *et al.*^23^ with permission from American Chemical Society.

Zhang *et al.* used FIB milling to fabricate two circular in-plane nanopores in a glass substrate (Fig. 7ai–vi).^85^ Two distinct FIB milling strategies were employed for nanopore fabrication. The first design used setting the FIB stage at 54° and drilling a lamella wall on the glass surface at 0° followed by changing the stage alignment to 0° and milling the nanopores at an incident angle of 36° (Fig. 7ai–iii).^151^ For the second design, the stage was tilted by 28° while the incident angle was set at 26° to construct a tilted lamella design (Fig. 7aiv–vi). The incident beam manipulation strategy and substrate orientation offered control over the final nanopore geometry. The vertical lamella design produced a conical shaped pore, while the tilted lamella formed a cylindrically shaped nanopore (Fig. 7aiii and vi).^151^

The same group later developed an in-plane nanopore sensor featuring three in-plane nanopores in-series, each with dimensions of 200 nm width, 200 nm depth, and 600 nm length as illustrated by SEM images in Fig. 7b.^152^ Similar to the previous design, this device integrated microscale regions fabricated *via* photolithography and wet chemical etching with nanoscale features fabricated *via* FIB milling. However, instead of creating a circular nanopore into a lamella wall, they applied a vertical incident beam at 90° perpendicular to the substrate surface to create nanofilters, nanochannels, and nanopores (Fig. 7c).^152^ Moreover, this sensor design added a 3rd nanopore to improve detection accuracy. As shown in Fig. 7d and e, translocation of one biological nanoparticle through the three in-plane nanopore sensor generated three consecutive current blockades.^152^ Each particle size was inversely proportional to the signal amplitude as predicted by eqn (5).^115,153,154^ The main limitation associated with these glass-based devices is the low device production rate; each device requires multiple techniques for micro/nanostructure fabrication including photolithography, etching, and FIB milling.

Harms *et al.*^155^ utilized a dual-nanopore configuration (two nanopores in series) spaced 2 μm apart and each nanopore was 50 × 50 × 40 nm in width, depth, and length (*W* × *D* × *L*) to analyze hepatitis B virus (HBV) capsids. The *μ*_app_ was calculated from the time difference (Δ*t*) between two consecutive RPS translocation events and reflects the vector sum of the EOF and the electrophoretic mobility (*μ*_ep_) of the particle. In a follow up study, this same group employed a similar sensor design but increased the nanopore length to 400 nm for size resolution enhancement. This modification allowed size-based discrimination of HBV capsids by analyzing their distinct ionic current blockade signatures.^18^

Kondylis *et al.*^51^ used in-plane nanopore devices containing two, four, or eight pores in series, spaced 500 nm apart to study the assembly of HBV capsids. The use of multiple pores supported enhanced precision, allowing the researchers to detect subtle differences in *μ*_app_ of capsids. Additionally, they were able to monitor the real-time changes in particle size and their relative abundances during the first hour of *in vitro* capsid assembly. In another capsid assembly study, Zhou *et al.*^19^ improved measurement precision *via* multicycle RPS in which each capsid was electrophoretically moved back and forth through four in-plane pores in series by alternating the polarity of the applied voltage. This allowed collecting ∼1000 individual RPS measurements, which lead to discriminating particles by size. They utilized the same method to investigate HBV capsid disassembly at varying concentrations of a chaotropic agent (guanidine hydrochloride).^156^ Furthermore, Li *et al.*^157^ employed four in-plane pores in series to determine that RNA length and structure, which effected capsid geometry during the assembly of Simian Virus 40 (SV40).

### Plastic (organic) in-plane nanopores and their surface modification

Over the past several years, plastics, in particular thermoplastics, have been utilized to make nanopores offering significant advantages in terms of fabrication and engineering surface chemistry. For example, in-plane nanopores can be fabricated using low cost and high throughput replication techniques, such as nano-injection molding.^158^ Plastics are robust materials that can tolerate different temperatures, pH, and salt conditions. Additionally, plastics exhibit diverse surface chemistries displaying high susceptibility to various surface activation, modification, and immobilization techniques.^159^

**Fig. 8:**
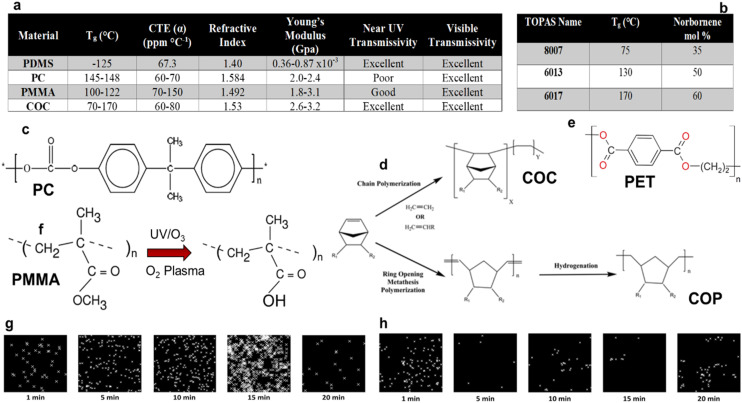
(a) Chemical, mechanical and thermal properties of various thermoplastics. CTE – coefficient of thermal expansion. (b) The glass transition temperature (*T*_g_) of the copolymer, COC, which consists of ethylene and norbornene units. The *T*_g_ depends on the mole fraction of norbornene in the copolymer. Chemical structures of different thermoplastics including PC (c), COC and COP (d) and PET (e). (f) Photo-oxidation of PMMA using either UV/O_3_ or O_2_ plasma to convert the methyl ester into surface-confined carboxylic acid groups. Reproduced from Chan *et al.*,^160^ with permission from Elsevier B.V. (g and h) Superresolution images of COC and PMMA, respectively, following UV/O_3_ photo-oxidation at different irradiation times. Reproduced from O'Neil *et al.*^161^ with permission from American Chemical Society.

Thermoplastics, are a class of polymers that consist of long chains of covalently linked repeating units with the chains held together by weak intermolecular forces. They are characterized by their ability to repeatedly undergo a phase transition either into a malleable form (*T* > *T*_g_; glass transition temperature) or a melt form (*T* > *T*_m_; melting temperature) and reshaped without undergoing significant chemical degradation. This unique property, stemming from their molecular structure, makes them susceptible to various manufacturing processes even at the nanoscale. Each type of thermoplastic has unique chemical, mechanical, and thermal properties (see Fig. 8a), which are determined by the chemical structure of the thermoplastic (Fig. 8c–e). However, these properties can be systematically altered using a copolymer, such as COC, which consists of ethylene and norbornene monomer units. As seen in Fig. 8b, the *T*_g_ of COC depends heavily on the mole fraction of norbornene in the copolymer.

Thermoplastics can be modified upon exposure to UV/O_3_ or an O_2_ plasma, which induces a photo-oxidation reaction that alters the surface chemistry of the plastic (see Fig. 8f).^160^ The primary modification is the formation of oxygen-containing groups including surface carboxyl groups that affects surface charge and wettability of the plastic. Characterization of these activated surfaces has been demonstrated.^162,163^ The surface charge and wettability can be controlled by dosing the plastic with different irradiation times. For example, using superresolution microscopy (STORM), which provides sub-diffraction limited spatial resolution, the distribution of –COOH groups on COC and PMMA were determined (see Fig. 8g and h).^161^ As seen from these superresolution images, the surface charge density was dependent on the dose of the activating radiation. Fig. 8g shows increases in –COOH density as COC substrates were exposed from 1 to 15 min of UV/O_3_ radiation after which a decrease in –COOH density was observed. Different effects were observed for PMMA (Fig. 8h), where the –COOH surface density was significantly less than COC and showed an abrupt decrease after 1 min of UV/O_3_ irradiation.

In terms of nanopore sensing, plastics do offer some unique advantages including the ability to manufacture in-plane pores using replication-based techniques that can improve the widespread use of RPS label-free detection, dielectric properties that can reduce high frequency noise, and the ability to easily modify their surfaces. However, there are some challenges including their high surface hydrophobicity in their native state (water contact angle >90° in some cases) that requires surface activation to make the nanostructures they contain more hydrophilic. Additionally, their amorphous nature creates non-homogeneous surface charge densities following activation (see Fig. 8g and h) that can give rise to nanobubble formation as well as create surface ionizable groups both of which can increase the 1/*f* noise in the RPS measurement.

### Thermoplastic in-plane nanopore sensors fabricated using replication technology

**Fig. 9:**
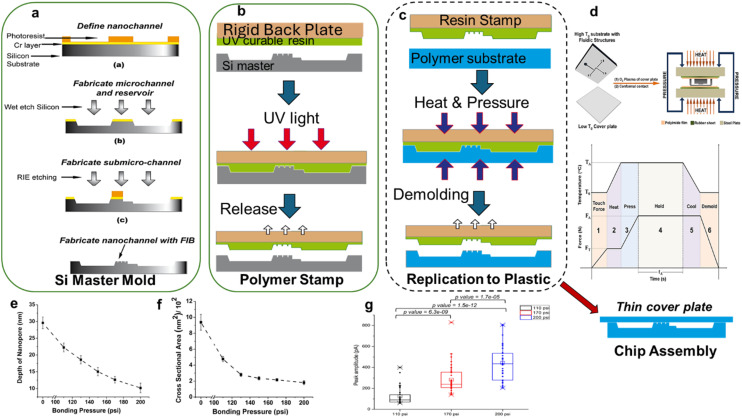
Diagram showing the general fabrication and assembly steps for making in-plane nanopore sensors in plastics. (a) Fabrication of the Si master mold, which utilized a combination of conventional photolithography to make the necessary microstructures followed by focused ion beam milling to produce the nanostructures, which included nanochannels and nanopores. (b) Fabrication of a resin stamp using UV-NIL with the Si master serving as the template. (c) Replication of the final device into a plastic using either thermal NIL or nano-injection molding. Reproduced from Shiri *et al.*^158^ with permission from The Royal Society of Chemistry. (d) Hybrid thermal fusion bonding of a low *T*_g_ cover plate to a higher *T*_g_ substrate containing the micro- and nanostructures. This step serves to enclose the fluidic channels and cover the in-plane nanopore. Reproduced from Uba *et al.*^164^ The Royal Society of Chemistry. The in-plane nanopores fabricated in the plastic could be reduced in size in a controllable manner during the thermal fusion bonding step, as shown in (e and f) in terms of the pore depth and overall cross-sectional area, respectively. (g) RPS peak amplitude for the translocation of λ-DNA (48.5 kbp) through plastic in-plane pores made in PMMA with a COC 8007 cover plate for three different devices assembled at different thermal fusion bonding pressures, 110, 170, and 200 psi. Reproduced from Athapattu *et al.*^52^ with permission from American Chemical Society.

One of the advantages of using thermoplastics is their ability to be molded into mixed-scale structures (nm → mm) using replication technologies such as nanoimprint lithography (NIL), hot embossing, or injection molding.^9,86,158,159,165^ The advantage of these approaches for nanopore fabrication is that Si masters can be prepared with the appropriate topology and then used as a master to generate molding tools for any of the aforementioned production processes. For example, in the case of nanopore sensors, a fluidic network can be generated in the Si master using conventional photolithography followed by FIB milling to produce the necessary nanostructures (hybrid lithography). Then, the Si master can be used to make resin stamps for prototyping or Ni molding tools for high-scale production (see Fig. 9a–c). There are several advantages of this approach: (i) any substrate material (*i.e.*, thermoplastic) can be used at the replication stage to suite the application need; (ii) different replication methods can be employed to produce the mixed-scale structures; (iii) no dependency on FIB or photolithography to make each device reducing cost and increasing production rate; (iv) ability to make mixed-scale structures to improve device figures-of-merit; and (v) integration of sample processing capabilities to the nanofluidic chip prior to making the RPS measurement.

Nanoimprint lithography (NIL) has been used to achieve high quality nanoscale imprints.^166–168^ This instrument is capable of imprinting sub-10 nm structures by accurately controlling the heat and pressure during device fabrication.^169^ Recently, nano-injection molding has been reported to produce high quality nanostructures.^158^ Nano-injection molding of nanosensors was demonstrated using resin stamps as a prototyping tool.^158^ The primary advantage of injection molding over NIL is that the production rate is higher and at a lower unit cost.^170^

A challenge associated with making nanofluidic devices using top-down strategies as outlined above is the need for enclosing the fluidic network with a cover plate. While there are several methods to bond a plastic cover plate to the substrate containing the fluidic network, the approach adopted must not damage the underlying nanostructures. The choice of thermoplastic as the cover plate depends on the type of experiment being conducted. If detection and identification of biomolecules involve spectroscopic analyses where the light should penetrate into the device and excite the targets followed by microscopic detection of the reporters, it's crucial to select a cover plate that exhibits excellent optical transparency. However, this is not an issue for RPS measurements where only electrical signals are used to analyze the target.

To minimize nanostructure deformation, a hybrid thermal fusion bonding technique was reported (see Fig. 9d),^164^ which consists of using a *T*_g_ cover plate thermal fusion bonded to a higher *T*_g_ substrate. This hybrid method has resulted in making nanopore sensor devices with a process yield rate >90%.

Recently, Athapattu *et al.* reported a technique to tailor the size of in-plane plastic nanopores as part of the thermal fusion bonding step;^52^ the goal was to increase the SNR for the RPS by reducing the size of in-plane nanopores made *via* replication. This was accomplished by increasing the bonding pressure while holding the temperature constant at 70 °C (*i.e.*, close to the *T*_g_ of a COC cover plate but below the *T*_g_ of the substrate).^52^ Increasing the pressure from 110 to 200 psi led to a reduction in the apparent diameter of the nanopore from 22.3 ± 1.4 nm to 10.2 ± 1.5 nm (inter-device standard deviations), an approximate 60% reduction (see Fig. 9e and f). The low standard deviations in these values indicated the high reproducibility of the fabrication method as well as the pore shrinkage process during thermal fusion bonding.

To demonstrate the ability to improve the SNR of the RPS measurements using this pore shrinking method, double-stranded DNA (λ-DNA) was electrically translocated through in-plane plastic nanopores (see Fig. 9g). The average peak amplitude of λ-DNA RPS events for devices bonded at 110 psi was 130 pA for λ-DNA corresponding to a pore depth of ∼22 nm as measured from conductance plots of 1 M KCl. Devices bonded at 170 psi had a pore depth of ∼13 nm and generated a RPS amplitude of 280 pA for λ-DNA and devices bonded at 200 psi possessed a depth of ∼10 nm and yielded a RPS amplitude of 437 pA for λ-DNA.

**Fig. 10:**
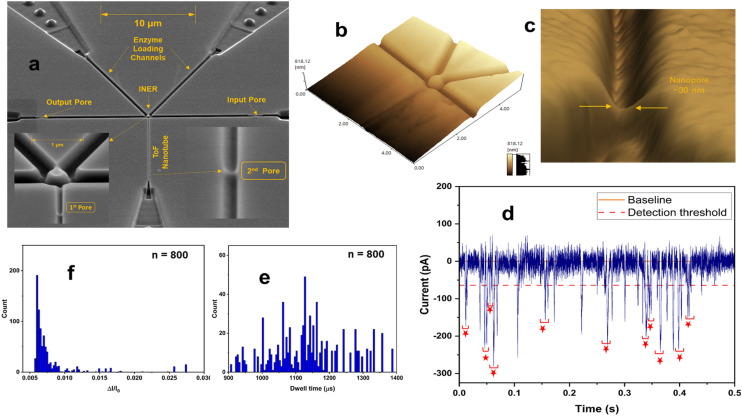
(a) Si master mold for preparing resin stamps for nano-injection molding. The nanofluidic circuit contains several functional components to allow for inputting single molecules into an immobilized nanoscale enzymatic reactor (INER) and monitoring products generated using the products in real-time using a dual in-plane nanopore sensor equipped with a ToF nanotube and two in-plane pores. (b) Atomic force microscopy (AFM) image of the INER. (c) AFM image of an in-plane pore (input pore; Fig. 5a). Both AFM images are of the nano-injection molded structures. (d) RPS data for a 100 nM solution of rCMPs in 1× NEBuffer 3 using the dual in-plane nanopore sensor. (e and f) Histograms of the dwell time (*t*_d_) and the normalized RPS peak amplitude (Δ*I*/*I*_0_), respectively. Reproduced from Shiri *et al.*^158^ with permission from The Royal Society of Chemistry.

Fig. 10 shows the results for an injection molded plastic nanofluidic device containing in-plane nanopore sensors integrated within a nanofluidic circuit allowing on-chip sample processing prior to performing the RPS measurement.^158^Fig. 10a displays the nanofluidic network fabricated in Si using both photolithography to define microstructures and FIB milling to construct the necessary nanostructures.^158^ The device structural dimensions ranged from ∼10 μm (microchannels) down to ∼30 nm (in-plane pores). AFM images of a solid-phase bioreactor (Fig. 10b) and an in-plane nanopore (Fig. 10c) are shown.^158^ The effective diameter of the in-plane nanopores in the assembled nanofluidic device, estimated using open pore current measurements, was ∼12 nm.^83,158^ Assembled COP/COC dual in-plane nanopore devices were tested with a rCMP solution (10 nM) in 1× NEBuffer 3 (pH 7.9) under an applied voltage of 2.5 V and RPS detection was carried out. Fig. 10d–f shows representative RPS data along with histograms of *t*_d_ and Δ*I*/*I*_0_.

The current blockade amplitude (Δ*I*) for a single-pore system is described by eqn (5) and demonstrated that Δ*I* is influenced by multiple parameters including the applied voltage, salt (electrolyte) type and concentration, pore size, and analyte size. Δ*I* scales proportionally with the hydrodynamic cross-section of the analyte and inversely proportional to pore volume indicating that smaller pores and larger analytes yield more prominent current blockades. For a dual in-plane nanopore sensor, the current modulation is more complex than the single-pore system. For such systems, Δ*I* can be given by eqn (8), which accounts for both nanopores and their contributions to the overall current modulation;^165^8
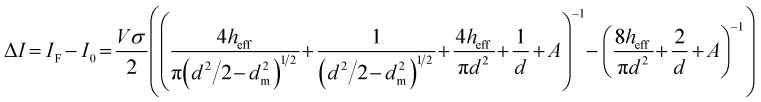
where *h*_eff_ is the effective pore length, *d* is the diameter of the pore (assuming both pores 1 and 2 in the series have similar geometry and dimensions), *I*_0_ is the open pore(s) current, *I*_F_ is the resultant RPS signal when only one pore is occupied, *σ* is the solution conductance, *d*_m_ is the diameter of the particle moving through the pore, and the term (*d*^2^/2 − *d*^2^_m_)^1/2^ corrects for the diameter of a semi-circular pore (U-shaped pore with a flat cover plate). Eqn (8) was derived based on assuming the following: (i) the nanochannel flight tube, positioned between two in-plane nanopores possesses higher resistance compared to the nanopore resistances due to its extended length, but its resistance is constant over the time (*i.e.*, it is not impacted significantly by the presence of a molecule); (ii) the electric field strength within the pores is larger than the flight tube and thus the majority of the resistive pulse signal is generated within the nanopores themselves; and (iii) the shape and size of the two pores in series are comparable. Eqn (8) still shows the dependency of the RPS signals on voltage, pore size(s), and solution conductance, similar to the single-pore system, but becomes more complicated due to the additional electrical components in series. Specifically, the total resistance in the fluidic network comprises the sum of the resistances of pore 1, pore 2 and the nanometer flight. Although the flight tube's contribution to the overall resistance is significantly high due to its longer length (*h*) and larger cross-sectional area, it does not produce a measurable RPS signal, as the translocating analyte does not induce sufficient perturbation in that region.

Thermoplastic nanopore sensors can be engineered to contain more than one sensing pore within a single device (see Fig. 10).^168^ The multi-pore format offers several advantages for not only single biomolecule detection, but can: (i) increase the accuracy of deducing RPS parameters;^86,171^ and (ii) inclusion of an RPS-independent parameter called the time-of-flight (ToF), which correlates with the *μ*_app_ of the molecule.^158,168^ Because the ToF is independent of the RPS signal, a new set of parameters can be used to alter its value without affecting the RPS dependent variables such as *t*_d_ and Δ*I*/*I*_0_. The parameter space to alter the ToF is large because it is an electrophoretic and/or chromatographic effect that can be altered by changing the wall chemistry in the flight tube, the length of the flight tube, applied voltage, or carrier electrolyte.

**Fig. 11:**
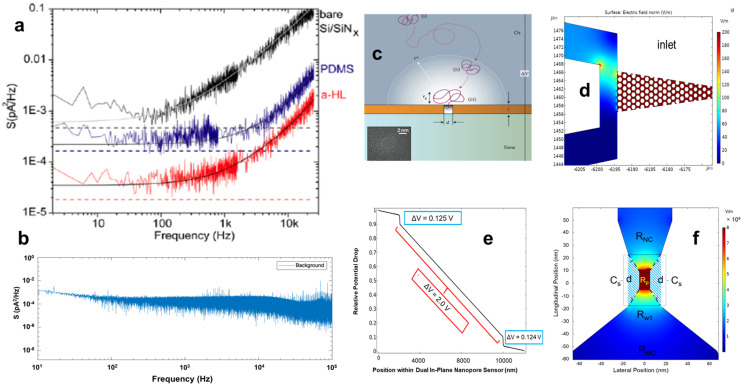
Noise characteristics from different types of nanopores. (a) Power spectral density functions for three different out-of-plane nanopores made in a SiN_*x*_ membrane (SSNP), PDMS (SSNP), and alpha hemolysin (biological NP). Taken from Tabard-Cossa *et al.*^56^ (b) Power spectral density of a COP/COP in-plane pore. Reproduced with permission from Shivanka *et al.*,^165^ copyright 2021 American Chemical Society. (c) Extension of electric field from an out-of-plane pore into the adjoining *cis*-chamber. Reproduced from Wanunu *et al.*^47^ with permission from Nature Nanotechnology. (d) COMSOL simulation showing extension of electric field across the pore traversing into the sampling microchannel. (e) Electric potential drop through a dual in-plane nanopore sensor, which is comprised of two in-plane pores in series and flanking a nanometer flight tube. (f) An in-plane nanopore showing the relative voltage drop at each longitudinal distance along the pore axis. *C*_s_ = capacitance of the polymer substrate (cross-hashed area; dot-dashed line shows capacitor plates, *d* = effective distance between plates), *R*_NC_ = nanochannel flight tube resistance, and *R*_MC_ = microchannel resistance. e and f Reproduced from Rathnayaka, *et al.*^86^ with permission from The Royal Society of Chemistry.

Fig. 11 shows the comparison of noise characteristics and electric field distribution of a dual in-plane nanopore sensor with those of traditional out-of-plane nanopore sensors. There are notable differences between a single out-of-plane nanopore and the dual in-plane nanopore system, particularly in their power spectral density profiles (see Fig. 11a and b). At higher frequencies, the noise observed for the dual out-of-plane system is mainly caused by capacitive and dielectric effects, whereas the in-plane configuration has reduced high-frequency noise.^56^ The use of thick and highly insulating dielectric substrates, such as a plastic, can minimize thermal voltage noise arising from the pore material's parasitic capacitance and dielectric loss tangent. Lee *et al.* were able to generate low RMS noise levels at sub-pA in *I*_0_ for PET pores of ∼1.5 nm diameter.^172^

Additional factors were proposed to explain the anomalous data shown in Fig. 11a and b: (i) the extended pore length; the in-plane pores are longer than biological pores like α-hemolysin resulting in longer dwell times. This phenomenon reduces the signal distortion during analyte translocation caused by BW limitations. (ii) The use of a low ionic strength carrier electrolyte. Typically, nanopore measurements are made in the presence of 1 M KCl; Smeets *et al.* reported lower ionic strength electrolytes (at low salt concentrations) can yield better RPS SNR for relatively larger pore sizes.^173^ (iii) The absence of surface protonation/deprotonation noise; plastic in-plane pores can be operated at a pH > p*K*_a_ of the surface carboxyl groups (p*K*_a_ ∼ 5.0), which can avoid protonation/deprotonation contributions to 1/*f* noise.^174^ (iv) The high mechanical stability; plastic in-plane pores offer greater mechanical stability compared to pores suspended on thin membranes that reduces 1/*f* noise.

The voltage drop for out-of-plane pores is almost exclusively across the pores while that is not the case for in-plane pores that are embedded within a nanofluidic network (Fig. 11c and d).^47^ This architecture allows in-plane pores to achieve higher sampling efficiency than their out-of-plane counterparts. This enhanced performance is due to the dual in-plane nanopore sensor placed within a mixed-scale fluidic network. For example, the dual in-plane nanopore sensor shown in Fig. 10 was able to detect as low as 3.9 fg of input rNMPs, while out-of-plane pores typically require about 1 μg of material to produce reasonable capture rates. This is predicated on the fact that the sampling zone (r*), where the electric field is sufficiently strong to electrokinetically drive molecules into the nanopore. The size of this zone can be calculated from eqn (9);^47^9
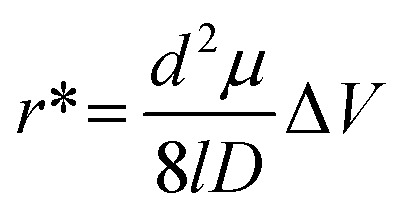
where Δ*V* is the voltage drop across the nanopore, *l* is the length of the pore, *d* is the effective diameter of the pore, *μ* is the electrophoretic mobility of the analyte, and *D* is the molecular diffusion coefficient of the analyte. For a molecule at a distance >r* from the pore, the motion to the pore is purely diffusive, and when the distance <r*, it becomes dominated by electrokinetic motion.^175^ As seen from the simulation result shown in Fig. 11d the effective electric field drop for a dual in-plane nanopore sensor extends into the sampling microchannel extensively unlike out-of-plane configurations. This extended electric field improves the sampling efficiency of in-plane pores.

Interestingly, despite the larger pore volume of the sensing in-plane pores relative to out-of-plane sensors, RPS detection of single nucleotides was successfully demonstrated using plastic in-plane pores while for an out-of-plane sensor required α-hemolysin pores modified with a β-cyclodextrin moiety to achieve similar detection efficiency. The in-of-plane pore volumes were 320 nm^3^*vs.* out-of-plane α-hemolysin pore volume that is ∼18.2 nm^3^.^176^ Considering only volume displacement effects (*i.e.*, Coulter principle), the detection of single nucleotides should be challenging using the in-plane nanopore system. However, the mixed-scale fluidic circuit, which consisted of nanochannels, and two in-plane pores placed in series, introduces additional physical effects which contribute to the resultant RPS signals. For example, Lee *et al.* demonstrated that Δ*I* for single-molecule RPS events can be significantly enhanced using a guiding nanochannel in series with the sensing nanopore due to compartmental limitations on ion transport as well as the drag forces exerted on the translocating molecules induced by the EOF in the guiding nanochannel.^177^ Furthermore, as depicted in Fig. 11e and f, the electric field strength in the dual in-plane nanopores are larger than the flight tube, but there is still an appreciable electric field drop within the nanochannel flight tube. This helps to suppress diffusional drift of the analytes and thus, minimize the variation in the ToF values.^86^

**Fig. 12:**
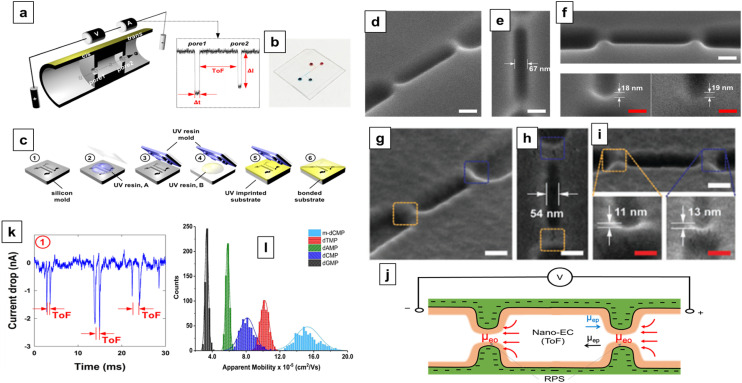
Thermoplastic-based fluidic device. (a) Schematically drawn set-up image of dual in-plane nanopore sensor, which consists of two in-plane nanopores flanking a nanometer-scale flight tube to allow carrying out nanoscale electrochromatography from which the ToF is deduced. The data includes the traditional RPS parameters (event amplitude and dwell time) and the ToF parameter. (b) Assembled sensor made from PEGDA. (c) Fabrication protocol used to make the device, which consisted of using UV-NIL from a Si master. (d–f) SEMs of the Si master mold including the dual in-plane pores flanking the flight tube, flight tube, and in-plane pores. (g–i) SEMs of NIL imprinted device made from PEGDA including the entire sensor, flight tube, and in-plane pores. Panels (a–i and k) were reproduced with permission from Choi *et al.*,^9^ copyright 2021 Wiley-VCH GmbH. (j) Schematic of sensor showing the two phases of the measurement including translocation through both pores and then the electrokinetic motion through the nano-EC flight tube. Within the flight tube, electrochromatography occurs and yields molecular-dependent ToF values due to differences in *μ*_app_ (apparent electrophoretic mobility). (k) RPS trace data for dCMP showing pairs of RPS events. (i) Histogram distributions of *μ*_app_ for the canonical dNMPs and m-dCMP collected by PMMA/COC (PMMA as substrate and COC as cover plate) device containing 100 μm nanochannel. Reproduced with permission from O'Neil *et al.*,^178^ copyright 2018 Elsevier B.V.

Choi *et al.* developed three different dual in-plane nanopore devices using PEGDA, each containing flight tubes with lengths ranging from 0.5–5.0 μm and were designed to improve nucleotide identification by enhancing differences in the ToF values (Fig. 12a–c).^9^ SEM images of the nanochannel flight tubes incorporated into the dual in-plane nanopore sensor are shown in Fig. 12d–i. After translocating individual deoxynucleotide monophosphates (dNMPs) through each device, the study revealed that identification accuracy for the four dNMPs improved from 55 to 94% with increasing flight tube length. The reason behind this improvement was ascribed to the combined effects of electrophoresis and chromatography in the nanometer sized flight tube (nano-EC). Because chromatographic separation resolution is column length dependent, longer flight tubes provide better separation in the ToF values.^9^Fig. 12k displays three pairs of current transients resulting from a single dNMP passing through the dual in-plane nanopore sensor.

The nano-EC of nucleotides within a nanofluidic channel was investigated by O'Neil *et al.*^178^ using laser-induced fluorescence detection. They electrokinetically transported dye-labeled dNMPs through 100 μm long nanochannels fabricated in PMMA/COC thermoplastics. As observed in Fig. 12i, the dNMP nano-EC migration order of nucleotides was identical to the results obtained by Choi *et al.*,^9^ who used RPS detection. The surface charges generated due to chemical groups (*e.g.*, carboxyl groups) on plastic walls either by O_2_ plasma or UV/O_3_ activation can influence ion migration, especially because diffusional distances are smaller in nanometer columns compared to microscale columns; thus, nano-EC effects are predominate (Fig. 12j).^30^ Additionally, surface activation increases the hydrophilicity and thus the wettability of the plastic surface, which is crucial for maintaining consistent EOF.^179^ When an electric potential is applied across a nanochannel, the diffuse layer near the surface charges drags the bulk fluid towards the cathode generating the EOF.^180^ The net *μ*_app_ of the analyte is a combination of the electrophoretic mobility (*μ*_ep_) and the mobility due to EOF (*μ*_eof_), which is further modified by solute–wall interactions.^181^

Altering O_2_ plasma or UV/O_3_ activation time affects *μ*_eof_ due to increases in the surface charge density within the nanochannel and can also act as a functional scaffold for surface modification using standard coupling chemistries. For example, Uba *et al.* chemically modified the surface of activated PMMA/COC nanochannels with ethylenediamine to the surface carboxy groups using EDC/NHS coupling chemistry.^182^ As a result, an inversion in the EOF was seen; 1.02 ± 0.017 × 10^−4^ cm^2^ V^−1^ s^−1^ for the carboxylated surface to −0.75 ± 0.021 × 10^−4^ cm^2^ V^−1^ s^−1^ for the diamine surface.^182^

**Fig. 13:**
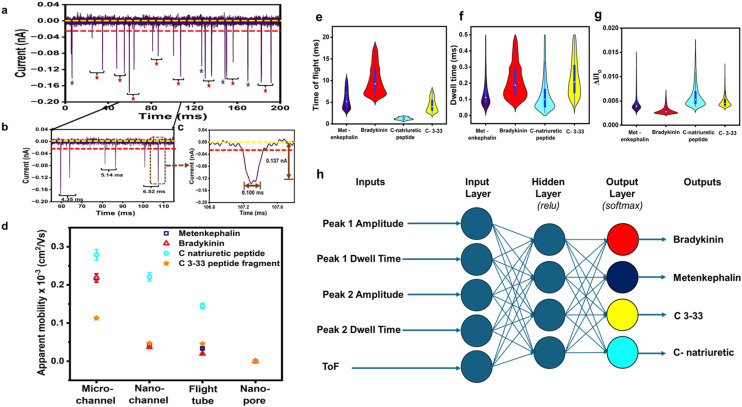
RPS data results acquired using series of peptides using dual in-plane nanopore sensor. (a) Current trace containing RPS events acquired by translocating metenkephalin peptide. (b) Paired events showing ToF values of the metenkephalin peptide. (c) Single event showing Δ*I*/*I*_0_ and peak dwell time. (d) The *μ*_app_ of four peptides measured specifically in the regions of microchannel, nanochannel and nanopore. Violin plot showing the distribution of ToF (e), dwell time (f), and Δ*I*/*I*_0_ (g). Neural network machine learning analysis of four peptides (h). Reproduced with permission from Chibuike *et al.*,^183^ copyright 2025 American Chemical Society.

Other molecule types can be detected and identified as well using the dual in-plane nanopore sensor. For example, Fig. 13 shows data using the dual in-plane nanopore sensor for detecting and identifying a series of peptides with the use of both the RPS and ToF parameters along with machine learning.^183^Fig. 13a–c display current transient signals collected at time intervals of 200 ms, 50 ms and 100 μs.^183^ These signals were generated by the peptide metenkephalin electrokinetically shuttled through the sensor using 2.5 V. Here, the dual in-plane nanopore chip was made from PMMA and contained a COC cover plate. The red dashed line represents the amplitude threshold used by the authors, which was set to keep the false positive rate to 0 in the blank. Inspection of Fig. 13d shows the apparent mobilities of four peptides (metenkephalin, bradykinin, C natriuretic and C 3-33 peptides) as they were electrokinetically transported through a microchannel, nanochannel, and nanopore.^183^ The authors observed that as the channel size was reduced, *μ*_app_ became smaller due to increased wall interactions.^183^ Smaller column lengths also reduced the differences in the ToF values. Although, the peptides did display differences in their ToF values (Fig. 13e), significant cluster overlap was observed when peptides were compared solely by their dwell times (Fig. 13f) and Δ*I*/*I*_0_ (Fig. 13g).^183^ Therefore, the authors implemented a neural network machine learning algorithm to improve the identification accuracy. Specifically, they used five input values (dwell time 1 and 2, Δ*I*/*I*_0_ 1 and 2 and ToF) to train the model and performed a validation test to determine the classification accuracy (Fig. 13h). As a result, they acquired 95.6% and 100% accuracy during training and validation tests, respectively.

## Comparison between in-plane *versus* out-of-plane nanopore sensors

Table 3 lists the performance metrics for both in-plane and out-of-plane nanopore sensors. Generally speaking there are advantages and disadvantages for both nanosensor platforms, but irrespective of configuration both offer single-molecule detection capabilities in a label-free manner and with the signal intensity inversely dependent on the size of the pore with respect to the target analyzed. The in-plane pores can easily be fine-tune in terms of the pore size and shape during ion beam milling or electron beam lithography. In addition, both in-plane and out-of-plane nanopore sensors can be made from organic or in-organic substrates. However, the out-of-plane pores can use biological nanopores while this becomes difficult for the in-plane format.

Comparison of the operational characteristics for in-plane and out-of-plane nanopore sensors

**Table:** 

Single molecule sensing by RPS			
LOD	<1 amol due to high e-field extension into sampling microchannel	>10 nmol due to abrupt size reduction (sampling reservoir to nanopore)	In-planes allow the use of gradient structure transitions while out-of-plane use a step transition
Bandwidth	High signal bandwidth 73/130 MHz (COP/PMMA)	Good signal bandwidth 16/32 MHz (SiN/PET)	In-plane have lower surface area capacitor plates
Signal integrity	Highly reproducible – depends on fabrication reproducibility	Highly reproducible for biological and modest for SSNP	For in-plane SSNP, milling conditions and pore closing are challenging
Operational time	Long if cover plate remains sealed to substrate	Modest due to instability of lipid bilayer for biological pores	Will see baseline drift (*i.e.*, changes in *I*_0_) over time for both systems
Noise characteristics	Low noise at high frequency due to low capacitance and dielectric of plastics	High noise at high frequency due to capacitance and dielectric considerations	Material selection and small capacitor plates for in-plane pores; out-of-plane pores are suspended within membranes
Multi-detection	Very simple due to planar nature of pore and fluidics	Challenging due to the non-transparency of membrane and alignment issues with vertical pore	Optical alignment issues become less problematic for in-plane pores, but must be careful with material selection (*i.e.*, optical transparency)

**Table:** 

Manufacturing			
Multiple pores (series or in-parallel)	Many pores can be placed in series or parallel (lithography dependent)	Pores in series determined by layer-by-layer fabrication; pores in parallel as well	In-plane pores can produce many (>8) in series or even in-parallel, but limited for out-of-plane pores for in-series
Material selection	High – organics (polymers), inorganics (Si-based)	High – biological, inorganic (SiN, SiO_2_, Al_2_O_3_), 2D materials (hBN, MoS_2_), organic (track-etched polymers)	No reported papers on using biological pores for in-plane configuration
			A variety of polymers are available for in-plane configuration
Surface modification	Activating the substrate using UV/O_3_ for plastics followed by covalent modifications	Site-directed mutagenesis for biological pores and UV/O_3_ for plastics	Plastic in-plane and out-of-plane pores can undergo similar activation and modification strategies
Manufacturing scalability	High-scale production at low cost when using thermoplastic as substrate	Low-scale production at high cost; CMOS production line can increase production rate	Top-down fabrication for in-plane pores and ion/electron beam milling for out-of-plane pores
Integration with sample pre-processing	Simple due to chip-format of in-plane pores	Difficult due to non-chip format of out-of-plane pores	Microchip-to-nanochip interfaces simple for in-plane pores
Multiple pores (series or in-parallel)	Many pores can be placed in series or parallel (lithography dependent)	Pores in series determined by layer-by-layer fabrication; pores in parallel as well	In-plane pores can produce many (>8) in series or even in-parallel, but limited for out-of-plane pores for in-series

In terms of noise characteristics, the in-plane pores can display higher mechanical stability compared to out-of-plane nanopore sensors, which are embedded within thin membranes. Also, capacitive noise for the in-plane pores is lower because the capacitor plates are smaller for these pore configurations. When the in-plane or even out-of-plane pores are made in a thermoplastic, this can make the dielectric noise smaller compared to SiN_*x*_ membranes.

When seeking to build multiple pores in series, this becomes a simple and more flexible process for in-plane pores compared to out-of-plane pores because the fluidic circuit can be laid out on a single plane and then fabricated using, for example, ion beam milling. In addition, the spacing between the pores is easily adjusted to allow for securing molecular characteristics of the target through the apparent electrophoretic mobility that can aid in molecule identification.

The particularly attractive operational characteristic of the in-plane pores is their ability to provide high sampling efficiency that can generate favorable mass LODs. This results from the size gradients in pore/channel dimensions that can produce high electric fields extending into micro-sampling channels as opposed to relatively large sample reservoirs that flank either side of a membrane containing the nanopore as is the case for out-of-plane nanopore sensors.

## Conclusions

Nanopore-based systems coupled with RPS have emerged as a powerful technique for the detection and identification of single molecules across a wide range of applications due to their ability to operate in a label-free manner and requiring rather simple instrumentation. Biologically derived nanopores such as α-hemolysin or MspA have significantly contributed to the progress of single molecule RPS sensing, but integrating them into broad user communities with long operational times is challenged due to the fragile nature of the lipid bilayer they are suspended in. This limitation has spawned the renaissance of SSNPs, which offer greater mechanical stability, improved fabrication versatility and broader flexibility in material selection, ranging from inorganic materials (Si and glass) to organic polymers (PET). In many cases, the nanopore systems employ a single pore configured in an out-of-plane format, where the analyte translocation is orthogonal with respect to the plane of the sensor. In these configurations, molecular identification primarily relies on conventional RPS parameters, namely the normalized current drop (Δ*I*/*I*_0_) and dwell time (*t*_d_). While attractive results have emanated from this approach, for example in the case of DNA/RNA sequencing, emerging applications increasingly demand more complex and information-rich datasets requiring additional parameters for single molecule identification. This is particularly true for the case of epitranscriptomic RNA modifications (>170 different modifications), and post-translational modifications (PTMs) in proteins, where subtle structural variations are challenging to resolve only with RPS parameters. Another limitation of out-of-plane nanopore sensors are their low mass loading efficiency. While amplification strategies can be envisioned to improve the sensitivity of nucleic acid detection, the amplification process can potentially mask epigenetic and epitranscriptomic modifications. The absence of amplification strategies for proteins further limits the detection sensitivity and characterization.

In-plane RPS nanopore sensors have recently emerged as a promising advancement. This platform offers double-sensing, where two spatially separated nanopores are connected *via* an intervening nanochannel. This geometry not only provides single-molecule detection in a label-free manner, but also facilitates ToF analysis, offering an additional single-molecule discriminatory parameter. The ToF is drawn from a combination of electrophoresis and chromatography with each molecule possessing a unique ToF value depending on its size and charge as well as its propensity to interact with the nanochannel wall.

Although glass-based in-plane nanopore sensors have been used to detect and identify a range of single molecule types, their low-throughput production and labor-intensive fabrication processes remain a challenge. In contrast, thermoplastic-based nanopore systems address these limitations by enabling low-cost and scalable fabrication, flexibility in device material selection, and highly sensitive RPS measurements. In addition, simple activation and surface modification strategies are available to further expand the application portfolio for these sensors. These advancements make thermoplastic nanopores a compelling alternative for next-generation molecular diagnostics and bioanalytical applications.

A main challenge with nanopore sensors is their wide-scale dissemination is their fabrication issues. Production of sub-100 nm or even sub-nm dimensions for sensing small objects such as the amino acids,^127^ require a combination of conventional photolithography and electron beam lithography or ion beam milling to make solid-state nanopores (in-plane or out-of-plane). While biological nanopores can ameliorate this issue, other pendant problems arise including mechanical stability and the limited operational lifetime of the sensor due to issues with the supporting lipid bilayer.^97^

Possible venues for increasing the access of academic labs to SSNPs (in-plane or out-of-plane) for research is through commercialization, soliciting support for small-scale production from foundries, or access to in-house fabrication resources. Shown in Table 4 is a cost-of-good SNR.

**Table 4:** Cost-of-good analysis for producing inorganic *versus* organic (plastics) nanopore sensors

Step	Lithography & FIB^a^	CMOS^b^	Injection molding		
	Cost/chip ($)		Cost/chip ($)		Step
	Nano	Nano	Micro	Nano	
*Optical mask*	$0.10 (4″–8 per wafer)		0.05	0.50	*Mold insert* c
*Photolithography*	2.0	0.50	0.50	0.50	*Molding* d
*Etching (DRIE)*	10	0.50	0.50	0.50	*Post-processing* e
*Material*	Silicon		0.25	0.25	*Polymer*
*Sputter coating, FIB milling, and assembly*	75	0.75	0.75	0.75	*UV exposure and assembly* f
*Chemicals*	0.75	0.25	0.25	0.25	*Chemicals*
*Labor*	2.25	0.75	0.75	0.75	*Labor*
**Total cost/chip**	90.10	87.10	3.05	3.50	**Total cost/chip**
**Production rate**	8 per day	1000 per day	960 per day		**Production rate** g
**Yield rate**	80%	70%	100%	90%	**Yield rate** h ^,^ i

a Costs for lithography/FIB steps taken from typical user fees for various foundries.

b Same processing steps as for (^1^), but production scaleup included.

c Tooling for producing a Ni mold insert using photolithography only for micromolding and photolithography and FIB for nano-molding.

d Amortization for 10 000 chips.

e Includes equipment usage ($0.40 per chip) and labor ($0.10 per chip).

f Labor for cleaning chip.

g Includes labor, cover plate material, equipment amortization for fusion bonding.

h Structure replication used as limiting step.

i Yield rate set by success of fusion bonding.

## Conflicts of interest

The authors declare no competing interests.

## Abbreviations

A/D/(ADC): Converter analog-to-digital converter
AFM: Atomic force microscopy
BW: Bandwidth (range of frequencies the modulated signal occupies)
COP: Cyclic olefin polymer
COC: Cyclic olefin copolymer
CTE: Coefficient of thermal expansion
DNA: Deoxyribonucleic acid
ssDNA: Single stranded DNA
dsDNA: Double stranded DNA
*λ*_D_
: Debye length (*λ*_D_) measure of the electric double layer thickness
EDL: Electrical double layer
EOF: Electroosmotic flow
EBL: Electron beam lithography
PTMs: Post-translational modifications
EV: Extracellular vesicles
EDC/NHS: 1-Ethyl-3-(3-dimethylaminopropyl)carbodiimide/*N*-hydroxysuccinimide
FIB: Focused ion beam
GNR: Graphene nanoribbon
HBV: Hepatitis B virus
ICR: Ion current rectification
HA: Hyaluronic acid
ICP: Ion concentration polarization
*I*–*V* curve: Plot of current response to applied voltage
LPF: Low-pass filter
MspA porin: Mycobacterial porins (cell membrane pore formed by beta-barrel protein of *Mycobacterium smegmatis*)
WT: Wild type (gene or phenotype as it occurs in nature)
NIL: Nanoimprint lithography (method of fabrication of nanometer scale that uses a mold for patterning of structures under controlled pressure and temperature)
NMPs: Nucleoside monophosphate
dNMPs: Deoxyribonucleoside monophosphate (building blocks of DNA)
rNMPs: Ribonucleoside monophosphates (building blocks of RNA) physical mold to stamp a pattern onto a material)
PC: Polycarbonate
PDMS: Polydimethylsiloxane
PET: Polyethylene terephthalate
PMMA: Poly(methyl methacrylate)
RNA: Ribonucleic acid
RPS: Resistive pulse sensing
SNR: Signal-to-noise ratio
SSNPs: Solid-state nanopores
STORM: Stochastic Optical Reconstruction Microscopy
TEM: Transmission electron microscopy
*T*_g_
: Glass transition temperature (temperature where a material transition between glassy/rigid to soft/soft/leathery state)
*T*_m_
: Melting temperature (temperature at which one half of a complex is disrupted, is a measure of thermal stability)
ToF: Time-of-flight (in the context of nanopore sensors, the term is used to define the time between the translocation of pore 1 and pore 2 in dual in-plane nanopore systems)
UV/O_3_: Ultraviolet light/ozone treatment (process based on ultraviolet light used to generate ozone molecules, which then oxidizes and cleans surfaces by breaking down organic contaminants or to generate oxygen functional groups in material surfaces such as polymers)
Zeta (*ζ*) potential: Defined as the electric potential at the shear surface of solid surfaces and colloidal particles at the particle–liquid interface
α-HL: Staphylococcal α-hemolysin (α-HL) a β-barrel pore-forming toxin
Δ*I*: Change in ionic current
*I*_0_
: Baseline ionic current (commonly referred as background current or open pore current)
*t*_d_
: Dwell time (residence time of an analyte inside or interacting with the nanopore)
*μ*_ep_
: Electrophoretic mobility (movement of the charge particles under applied electric field)
*μ*_eof_
: Electroosmotic mobility (movement of the bulk solution)
*μ*_app_
: Apparent mobility which is the sum of electroosmotic mobility and electrophoretic mobility
*η*
: Viscosity of buffer solution
*ε*_o_
: Permittivity of free space (F m^−1^)
*ε*_r_
: Relative permittivity
Δ*V*: Voltage drop
*Cis* side: In nanopore sensor context refers to the side of the membrane where the analyte is added
*Trans* side: Opposite side of the membrane pore where the analyte translocates to

## Data Availability

All data associated with this manuscript is detailed in the form of figures and tables included in the manuscript. The data presented in all figures and tables can be made available to the readership upon request to the corresponding author of this manuscript.
